# Proteogenomic Profiling of Idiopathic Pulmonary Arterial Hypertension Identifies Sex‐Differential Proteins and Candidate Therapeutic Targets

**DOI:** 10.1002/advs.77687

**Published:** 2026-09-16

**Authors:** Xinjie Lin, Yangchang Zhang, Yang Pu, Duanke Liu, Tao Lei, Kai Ma, Qiyu He, Zheng Dou, Yuze Liu, Dengyuan Liu, Yinge He, Yanshang Wang, Xiaojiao Zheng, Yanbing Ma, Jianrong Zhou, Weiding Zhai, Binbin Su, Li Chen, Shoujun Li

**Affiliations:** ^1^ Pediatric Cardiac Surgery Center State Key Laboratory of Cardiovascular Disease National Clinical Research Center for Cardiovascular Diseases Fuwai Hospital, Chinese Academy of Medical Sciences & Peking Union Medical College/National Center for Cardiovascular Diseases Beijing China; ^2^ Department of Rheumatology and Immunology The First Affiliated Hospital of Chongqing University of Chinese Medicine Chongqing China; ^3^ School of General Education Chongqing College of Traditional Chinese Medicine Chongqing China; ^4^ Beijing Institute of Ophthalmology Beijing Tongren Eye Center Beijing Tongren Hospital Beijing Key Laboratory of Intelligent Diagnosis Technology and Equipment for Optic Nerve‐Related Eye Diseases, Capital Medical University Beijing China; ^5^ State Key Laboratory of Common Mechanism Research for Major Diseases Department of Biochemistry Institute of Basic Medical Sciences Chinese Academy of Medical Sciences and Peking Union Medical College Beijing China; ^6^ Zhengzhou No. 7 People's Hospital Zhengzhou China; ^7^ Division of Psychiatry Faculty of Brain Science University College London London UK; ^8^ College of Science China Agricultural University Beijing China; ^9^ School of Population Medicine and Public Health Chinese Academy of Medical Sciences/Peking Union Medical College Beijing China; ^10^ Institute of Child and Adolescent Health School of Public Health Peking University Beijing China

**Keywords:** biobank, drug repositioning, epidemiology, idiopathic pulmonary arterial hypertension, proteomics

## Abstract

**Background:**

Current biomarkers for idiopathic pulmonary arterial hypertension (IPAH) lack disease specificity and provide limited biological insight. We hypothesized that integrating population‐based proteomics with genetic evidence would identify robust IPAH‐associated proteins relevant to risk stratification, early detection, prognosis, sex differences, and candidate drug‐repurposing opportunities.

**Methods:**

We analyzed 2,918 plasma proteins in 44,137 predominantly European‐ancestry UK Biobank participants aged 40–69 years, including 252 incident and 141 prevalent IPAH cases. Proteins associated with IPAH were identified using Cox and logistic regression analyses, prioritized by cis‐Mendelian randomization, and assessed through cross‐ancestry effect‐direction concordance and sensitivity analyses. Unsupervised clustering, nested cross‐validated prediction models, and target–drug annotation were used for downstream evaluation.

**Results:**

Eighteen proteins showed convergent epidemiological and genetic evidence, and 12 remained supported in cross‐ancestry and sensitivity analyses. These proteins defined a high‐mortality endotype characterized by vascular and ventricular remodeling. Combined clinical and proteomic models achieved AUCs of 0.779 for incident IPAH and 0.768 for mortality. Proteomic contributions differed by sex, and target–drug annotation identified six drug‐linked proteins, highlighting ANXA2‐linked *Artenimol* as a repurposing candidate.

**Conclusions:**

Twelve biologically plausible IPAH‐associated proteins inform risk stratification, prediction, sex‐related characterization, and therapeutic prioritization. Independent multi‐ancestry validation and mechanistic studies are warranted.

## Graphical abstract

An integrated proteogenomic analysis of 44,137 predominantly European‐ancestry UK Biobank participants aged 40–69 years identifies 12 robust proteins associated with idiopathic pulmonary arterial hypertension. These proteins define a high‐mortality molecular endotype, support early detection and mortality prediction, reveal sex‐differential proteomic contributions, and highlight potential therapeutic targets and candidate drugs.

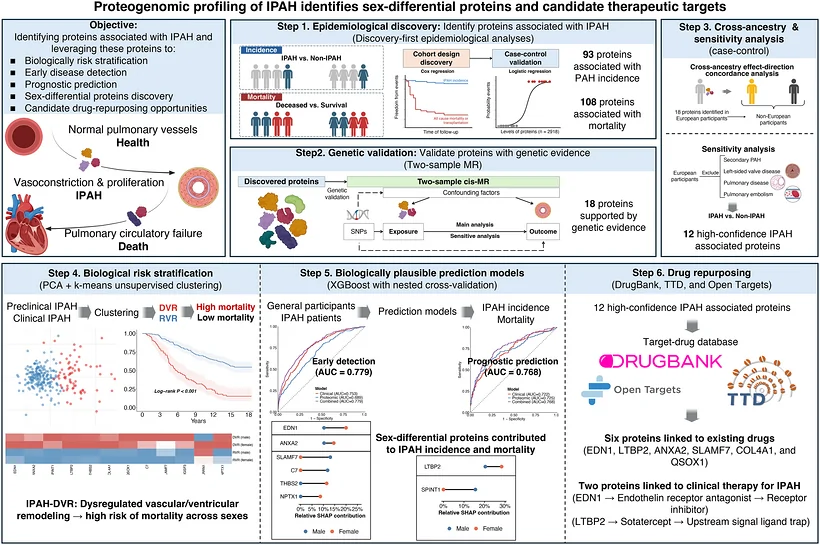

## 1 Introduction

Idiopathic pulmonary arterial hypertension (IPAH) is a rare but devastating cardiovascular disease, characterized by progressive pulmonary vasculopathy and secondary right ventricular (RV) failure [1]. Despite therapeutic advances, IPAH's prognosis remains poor, with a case‐fatality rate exceeding 10% annually [2]. Current guidelines underscore circulating biomarkers as indispensable tools for prognostic assessment [3, 4]. B‐type natriuretic peptide (BNP) and N‐terminal prohormone of BNP (NT‐proBNP) represent the established biomarkers for risk stratification in clinical practice [3, 4]. However, their utility is constrained by considerable susceptibility to confounders, such as age, obesity, inflammation, renal function, and cardiovascular comorbidities [5, 6, 7, 8, 9]. Moreover, as myocardial strain‐responsive indicators rather than primary drivers of pulmonary vascular lesions, their indirect and delayed reflection of vascular pathology hinders their utility for preclinical detection [10].

Circulating proteins, as functional molecules of intercellular signaling, offer a dynamic window into the active processes of pulmonary vascular remodeling [11]. However, translating proteomic signatures into clinical utility has been limited by methodological constraints. Most prior studies relied on modest epidemiological associations derived from disease‐specific cohorts, posing critical challenges [12, 13, 14, 15]: First, the marked heterogeneity in mortality risk inherent to IPAH is difficult to reconcile within clinical‐stratification frameworks. Second, registry studies fail to capture the preclinical status, leaving the early biomarkers of IPAH incidence largely unknown. Third, IPAH exhibits a profound epidemiological sex difference, where males exhibit lower susceptibility but higher mortality [16, 17, 18, 19], yet the proteomic correlates of this pattern remain poorly characterized. Finally, although current therapies primarily target the endothelin, nitric oxide, and prostacyclin pathways, as well as transforming growth factor‐β (TGF‐β) pathways, residual morbidity and mortality persist, underscoring the need for the development of additional therapeutic strategies for IPAH. In this context, we hypothesized that identifying IPAH‐associated proteins would aid in risk stratification, early detection, prognostication, understanding sex‐differential biomarkers, and candidate drug‐repurposing opportunities.

Addressing these gaps requires a transition from observational studies to biological inferences. A systematic integration of prospective epidemiology with genetic instrumentation provides a robust framework to identify true and translational signals [20]. Here, we developed a systematic proteomic investigation to identify robust IPAH‐associated proteins with potential translational implications.

## 2 Methods

### 2.1 Study Population

The UK Biobank received ethical approval from the Northwest Multi‐center Research Ethics Committee (16/NW/0274), and all participants provided written informed consent. All procedures involving human participants were conducted in accordance with the principles of the Declaration of Helsinki [21]. This study was conducted under the UK Biobank application number 105435.

The UK Biobank is a large‐scale, population‐based prospective cohort study that recruited over 500,000 participants aged 40 to 69 years, predominantly of European ancestry, from 22 assessment centers across the United Kingdom between 2006 and 2010. Herein, we selected 54,219 participants with available plasma proteomic data from the UK Biobank Pharma Proteomics Project (UKB‐PPP) Consortium. Participants with more than 50% missing protein data were excluded. Following this quality control, 53,004 participants were retained for subsequent participant selection and cohort derivation (Appendix S1).

### 2.2 Proteomic Measurement and Data Processing

The UKB‐PPP was established to generate a large‐scale plasma proteomic resource for genetics‐guided drug discovery, biomarker identification, and precision medicine research. For this study, we used existing UKB‐PPP proteomic data. Specifically, EDTA‐plasma samples were collected from UK Biobank participants at baseline assessment visits. Following central laboratory processing, plasma aliquots were stored in the UK Biobank automated −80°C archive and shipped on dry ice to the Olink Analysis Service in Sweden for proteomic profiling. Proteomic measurements were generated using the antibody‐based proximity extension assay with next‐generation sequencing readout on the Olink Explore 1536 and Expansion platforms, quantifying a total of 2,923 unique proteins across 2,941 assays arranged in four 384‐plex panels. Three proteins with missing values in more than 50% of participants were excluded. Protein abundances were expressed as log2‐transformed Normalized Protein eXpression (NPX) values to enable standardized comparison. NPX values with missing entries were imputed using a chained random forest algorithm that incorporated age and sex as predictors, and the resulting values were subsequently standardized to a standard normal distribution with a mean of 0 and a standard deviation of 1. Given the established prognostic value of BNP and NT‐proBNP in IPAH, we treated them as separate baseline variables, yielding a final proteomic set of 2,918 proteins.

### 2.3 Idiopathic Pulmonary Arterial Hypertension

In the UK Biobank, participants with IPAH were recruited from the general population rather than from specialized pulmonary hypertension centers or pulmonary hypertension registries. IPAH was identified from linked hospital inpatient and primary care data, corresponding to the International Classification of Disease, 10th Revision (ICD‐10) code I27.0, which, in principle, denotes a primary pulmonary hypertension phenotype excluding other Group I pulmonary arterial hypertension with identifiable etiology and secondary pulmonary hypertension classified into Group II to IV. To further minimize potential diagnostic overlap, individuals with ICD‐10 code I27.2 for secondary pulmonary hypertension were excluded. Additionally, to mitigate potential misclassification arising from ICD‐based disease definitions, conditions associated with Group I pulmonary arterial hypertension and Group II to IV pulmonary hypertension were excluded using multiple ICD‐10 codes in the sensitivity analysis (Appendix S2).

### 2.4 Participants Selection

The 53,004 participants with available measurements for 2,918 proteins were further screened to derive analytical cohorts. To minimize potential bias arising from population stratification, we excluded participants without a self‐reported European ethnic background (*n* = 3,583). Also, non‐randomly recruited participants (*n* = 4,997) were excluded to mitigate potential selection bias; those with prior lung transplantation or heart‐lung transplantation at baseline (*n* = 3) were excluded because transplantation represents one of our study outcomes; and participants diagnosed with secondary pulmonary hypertension (*n* = 284) were excluded to restrict the cohort to an IPAH‐compatible population. Thereafter, the remaining 44,137 UK Biobank participants constituted the overall analytic cohort, including 141 participants with IPAH at baseline, 252 participants who developed IPAH during follow‐up, and 43,744 participants without a recorded diagnosis of IPAH (Appendix S1).

### 2.5 Cohort Derivation

In the primary cohorts, participants without a recorded diagnosis of IPAH at baseline were assigned to the incidence cohort (*n* = 43,996) and followed for incident IPAH; whereas those diagnosed with IPAH at baseline were assigned to the mortality cohort (*n* = 141) and followed for all‐cause mortality, lung transplantation, or heart‐lung transplantation. Within each of the incidence and mortality cohorts, participants were further stratified by sex to establish sex‐stratified subcohorts (Appendix S1).

Additionally, we constructed a cross‐ancestry cohort using participants of non‐European ancestry excluded from the primary cohorts. These ethnic minorities were further filtered by excluding individuals with missing ethnicity data (*n* = 264), non‐randomized participants (*n* = 692), and those with concomitant secondary pulmonary hypertension (*n* = 17). Ultimately, the cross‐ancestry cohort comprised 2,610 ethnic minorities, including 26 with IPAH and 2,584 without a recorded diagnosis (Appendix S3).

To address potential misclassification from the ICD‐based case definition, we derived a more restrictive sensitivity cohort from the primary analytical cohort of 44,137 European participants by additionally excluding those with comorbid underlying conditions associated with Group I to IV pulmonary hypertension. These conditions included congenital heart disease (*n* = 124), HIV infection (*n* = 16), connective tissue disease (n = 265), portal hypertension (*n* = 20), hereditary hemorrhagic telangiectasia (*n* = 5), left‐sided valve disease (*n* = 242), chronic obstructive pulmonary disease (*n* = 260), interstitial lung disease (*n* = 5), obstructive sleep apnea (*n* = 186), and pulmonary embolism (*n* = 150). Accordingly, the final sensitivity cohort comprised 238 participants with IPAH and 42,626 controls without IPAH and was subsequently stratified by sex (Appendix S4).

For downstream clustering analysis, we included both the IPAH preclinical cohort (*n* = 252), consisting of participants without IPAH at baseline but diagnosed during follow‐up, and the clinical IPAH cohort (*n* = 141), comprising those with IPAH at baseline. For prediction models, the incidence prediction cohort included all participants without IPAH at baseline (*n* = 43,996), whereas the mortality prediction cohort included all IPAH patients (*n* = 393), comprising the preclinical cohort (*n* = 252) and the clinical cohort (*n* = 141) (Appendix S1).

### 2.6 Covariates

Covariates were obtained from the UK Biobank baseline assessments and included demographic, clinical, lifestyle, comorbidity, laboratory biomarker, and physical activity data. Demographic and clinical variables included age (continuous, years), sex (binary, male or female), body mass index (BMI; continuous, kg/m^2^), systolic blood pressure (continuous, mmHg), and diastolic blood pressure (continuous, mmHg). Lifestyle factors comprised smoking status (binary, yes [current/former] or no) and drinking status (binary, yes [current/former] or no). Comorbidities were identified using ICD‐10 codes [22], with corresponding codes presented in brackets: hypertension (binary, yes [I10‐I15] or no), coronary artery disease (binary, yes [I25] or no), heart failure (binary, yes [I50] or no), atrial fibrillation (binary, yes [I48.x] or no), diabetes mellitus (binary, yes [E10‐E14] or no), and chronic kidney disease (binary, yes [N18.x] or no). Conditions associated with secondary pulmonary hypertension were also recorded as baseline comorbidities in the primary cohort and were considered as exclusion criteria in the sensitivity analysis. Laboratory biomarkers encompassed alanine transaminase (ALT; continuous, U/L), creatinine (continuous, umol/L), C‐reactive protein (CRP; continuous, mg/L), glucose (continuous, mmol/L), glycated hemoglobin (HbA1c; continuous, mmol/mol), low‐density lipoprotein cholesterol (LDL; continuous, mmol/L), triglycerides (continuous, mmol/L), BNP (continuous, NPX), and NT‐proBNP (continuous, NPX). Physical activities were selected as subjectively reported walking pace (ordinal, slow pace, steady average pace, and brisk pace), total metabolic equivalent of tasks (METs) per week (continuous), and objective forced vital capacity (FVC; continuous, L).

IPAH‐specific measures, including direct assessments of hemodynamics, World Health Organization (WHO) functional class, six‐minute walk distance, and RV function, were unavailable owing to the absence of right‐heart catheterization and specialist pulmonary hypertension assessment in the population‐based UK Biobank dataset.

### 2.7 Study Outcomes

The outcomes were analyzed as time‐to‐event variables. Follow‐up commenced at each participant's enrollment (i.e., UK Biobank baseline assessment visit, 2006 to 2010) and terminated at the first occurrence of an outcome event or the prespecified administrative end of follow‐up on December 1, 2024, whichever occurred first. In the IPAH incidence cohort, the primary endpoint was the incidence of ICD‐10‐coded IPAH; in the mortality cohort, the primary endpoint was defined as all‐cause mortality, lung transplantation (Office of Population Censuses and Surveys Classification of Interventions and Procedures, version 4, OPCS‐4, E53), or heart‐lung transplantation (OPCS‐4, K01).

### 2.8 Proteomic Epidemiological Analyses

Two‐step cross‐validated epidemiological analyses were performed to discover robust plasma proteins associated with outcomes. First, we performed propensity score matching (PSM) using nearest‐neighbor matching without replacement, based on propensity scores estimated by logistic regression. The propensity score model included all prespecified baseline covariates described above. Participants were matched at a 1:4 ratio for the incidence cohort, yielding 252 incident IPAH cases and 1,008 matched controls, and a 1:1 ratio for the mortality cohort, yielding 60 mortality cases and 60 matched survivors. These ratios were chosen by weighing the availability of eligible controls against the marginal gain in statistical precision from additional matches. Covariate balance was evaluated using standardized mean differences (SMDs), with an absolute SMD < 0.1 considered acceptable; residual imbalances were further adjusted for in the models, prioritizing clinically relevant covariates with the largest remaining imbalance to avoid overfitting. Then, multivariable Cox regression models were used in the matched cohorts for protein discovery, and multivariable logistic regression was applied in a case–control framework to validate the discovered proteins. Each protein was modeled as an independent variable, adjusting for imbalanced covariates. To account for false‐positive findings arising from multiple testing, a false discovery rate (FDR) adjusted two‐sided *p*‐value < 0.05 (Benjamini–Hochberg method) was considered significant. Proteins were retained as discovery‐stage findings only if they were significant in both methods and showed consistent effect directions. Moreover, sex‐stratified protein discovery was conducted within the same framework, and a nominal two‐sided *p*‐value < 0.05 was considered a suggestive association. This liberal threshold was set to preserve sufficient discovery power after sex stratification.

### 2.9 Enrichment Analyses

To elucidate the biological pathways and functions of IPAH‐associated proteins, enrichment analyses were performed using the Gene Ontology (GO), Kyoto Encyclopedia of Genes and Genomes (KEGG), and Reactome databases. To ensure statistical rigor, these analyses were conducted against a given background reference set comprising the 2,918 proteins profiled in our study.

### 2.10 Proteomic Cis‐Mendelian Randomization (MR) Analysis

To assess whether the epidemiologically identified proteins were supported by genetic evidence, two‐sample cis‐MR analyses were conducted. Genetic instruments were constructed from cis‐acting protein quantitative trait loci (cis‐pQTLs) identified in 35,571 participants of European ancestry from the UK Biobank [23] (Olink platform), 35,559 Icelandic participants from the deCODE study [24] (SomaScan v4 platform), and 10,708 European‐descent participants from the Fenland study [25] (SomaScan v4 platform). For each protein, all single‐nucleotide polymorphisms (SNPs) located within ± 500 kb of the protein‐coding gene and associated with protein levels at genome‐wide significance (*p*‐value < 5 × 10^−^^8^) were selected as genetic instruments. To ensure instrument independence, variants in linkage disequilibrium were pruned at r^2^ < 0.001 using European reference data from Phase 3 of the 1000 Genomes Project.

Summary statistics for the outcome of diagnosed IPAH were obtained from genome‐wide association studies (GWAS) conducted in participants of Finnish ancestry from the FinnGen release 11 [26] (R11, finn‐b‐I9_HYPTENSPUL of 125 cases vs. 162,387 controls; finn‐b‐I9_HYPTENSPUL_EXNONE of 125 cases vs. 218,667 controls) and a previous meta‐analysis of participants of European ancestry [27] (GCST007228 of 2,085 cases vs. 9,659 controls).

The two‐sample MR analyses were performed for proteins with at least three independent significant SNPs. The inverse‐variance weighted (IVW) method was employed as the primary estimator, with Maximum Likelihood, MR‐Egger, and Weighted Median as supplementary approaches. Associations between plasma protein levels and IPAH risk were considered statistically significant at a nominal two‐sided *p*‐value < 0.05 within the IVW method. All analyses were restricted to individuals of European ancestry.

Three‐step sensitivity analyses for cis‐MR results were conducted to account for heterogeneity and directional horizontal pleiotropy and to obtain outlier‐robust estimates: (i) heterogeneity was assessed using I^2^, and proteins with substantial heterogeneity (I^2^ ≥ 50%) were excluded; (ii) directional horizontal pleiotropy was evaluated using the MR‐Egger intercept for analyses with at least three instrumental variables, and results with a significant intercept (*p*‐value < 0.05) were excluded to ensure the robustness of causal estimates. (iii) Outlier‐robust estimation required proteins to show directionally consistent effects between IVW and supplementary methods. Proteins failing these sensitivity criteria were excluded.

### 2.11 Protein‐Protein Interaction Analysis

Protein–protein interaction analysis was performed using the STRING database (*Homo sapiens*, https://string‐db.org) to explore known and predicted functional connectivity among the prioritized proteins. Interactions were derived from curated databases, experiments, co‐expression, text mining, gene neighborhood, gene fusion, and gene co‐occurrence and retained at a minimum interaction confidence score of 0.4. This exploratory network analysis characterized connectivity among individual proteins and complemented enrichment analyses of overrepresented pathways and biological processes.

### 2.12 Cross‐Ancestry Effect‐Direction Concordance Analysis

To assess the concordance of protein association directions across ancestries, we performed an exploratory case–control analysis in an independent non‐European subset. Given the substantially reduced case number, no formal statistical significance threshold was applied. The case‐control models were fit with logistic regression and adjusted only for age and sex. Proteins showing a consistent direction of effect with the European cohort and meeting the post hoc exploratory one‐sided *p*‐value < 0.2 was considered to provide suggestive cross‐ancestry support.

### 2.13 Sensitivity Analysis

To reduce misclassification arising from ICD‐based case ascertainment, we conducted a sensitivity analysis using a more stringent IPAH definition. Specifically, we excluded participants with etiologies associated with Group I pulmonary arterial hypertension and diseases linked to Group II to IV pulmonary hypertension, conservatively treating these conditions as potential causes rather than simple comorbidities. We applied the PSM with a 1:10 ratio, followed by case‐control analysis using logistic regression in both sexes and sex‐stratified cohorts. Proteins with a nominal two‐sided *p*‐value < 0.05 were considered to have passed sensitivity analysis.

### 2.14 Clustering Analysis

To identify biologically plausible endotypes associated with high mortality risk in IPAH patients, we performed unsupervised clustering of preclinical and clinical IPAH patients using high‐confidence proteins. Principal component analysis (PCA) was first applied to reduce the proteomic dimensionality, retaining the first two principal components for clustering. Then, optimal clusters were determined using the elbow and silhouette methods, and IPAH patients were grouped using the k‐means algorithm, which iteratively assigns each participant to the nearest centroid, updates centroids to the mean of all points in each cluster until convergence, and minimizes the total within‐cluster sum of squares. Moreover, we plotted Kaplan–Meier survival curves stratified by sex and endotype to compare cluster‐based risk stratification for mortality and assessed differences using the log‐rank test. To visualize endotype‐associated proteomic signatures and their sex‐specific patterns, we generated heatmaps of mean protein z‐scores (scaled from −1 to 1) across sexes and endotypes, as well as individual‐level protein expression heatmaps.

### 2.15 Biologically Plausible Prediction Models

To improve the prediction of IPAH outcomes and to assess sex‐differential proteomic contributions, we developed eXtreme Gradient Boosting (XGBoost)‐based, sex‐stratified prediction models using clinical, proteomic, and combined (i.e., both clinical and proteomic) variables. IPAH incidence prediction was developed in participants without IPAH at baseline, and mortality prediction was applied among all IPAH patients. Given the shortage of disease‐specific assessments in the population cohort, clinical predictors were selected to approximate the simplified ESC risk stratification framework, including general indicators (i.e., age, sex, BMI, systolic blood pressure, smoking status, drinking status, ALT, creatinine, CRP, HbA1c, LDL, and triglycerides), BNP, NT‐proBNP, and physical capability (i.e., walking pace, total METs per week, and FVC). However, hemodynamic assessments for IPAH was not included in model training because these indicators were not routinely collected across the UK Biobank cohort. Proteomic predictors were selected from identified high‐confidence proteins. Additionally, we employed a nested cross‐validation framework to improve the robustness of performance estimation and mitigate overfitting, which consisted of a 5‐fold outer loop for independent performance evaluation and a 5‐fold inner loop for feature selection (via LASSO regression) and hyperparameter tuning. Model discrimination was assessed using the area under the receiver operating characteristic curve (AUC) and compared using paired DeLong tests. Model calibration was assessed using calibration plots, calibration intercepts, calibration slopes, and Brier scores. The potential clinical utility of the mortality models was evaluated using decision curve analysis. The incremental value of the protein panel beyond BNP/NT‐proBNP was also quantified using the change in AUC and paired DeLong tests. For biological interpretability, a final consensus model was retrained on the entire dataset using the optimal hyperparameters identified during cross‐validation. SHapley Additive exPlanations (SHAP) values were then calculated across the full cohorts to quantify each protein's global contribution. The relative importance of these proteins for both sexes was visualized using dumbbell plots.

### 2.16 Potential Therapeutic Targets

High‐confidence proteins associated with IPAH were considered potential therapeutic targets. Because functional interactions among proteins may also influence biological effects, the target space was further expanded to include functionally connected receptors, ligands, or interacting proteins. To systematically identify drugs acting on these targets, three curated drug–target databases: DrugBank (https://www.drugbank.ca), Therapeutic Target Database (TTD, https://db.idrblab.net/ttd/), and Open Targets (https://platform.opentargets.org) were queried. All protein targets, drugs, drug groups, pharmacologic actions, and clinical indications were manually curated to support drug repurposing analyses and future therapeutic development.

### 2.17 Statistical Analysis

All statistical analyses and data visualizations were performed in R (version 4.2.2). Continuous variables were presented as means (standard deviations) and compared using a *t*‐test. Categorical variables were presented as counts (percentages) and compared using the Chi‐square test. Baseline tables were summarized using the *tableone* package (version 0.13.2). Using the *mice* package (version 3.18.0), missing NPX values for proteins were imputed using a chained random forest algorithm that included age and sex as predictors, and missing values in baseline covariates were imputed using random forest‐based multiple imputation. PSM was performed using the *MatchIt* package (version 4.7.2) to balance baseline covariates between groups. Multivariable Cox proportional hazard models were applied for protein discovery, and multivariable logistic regression models were conducted for cross‐method validation. Enrichment analyses were implemented using the *clusterProfiler* (version 4.18.2), *org.Hs.eg.db* (version 3.22.0), and *ReactomePA* packages (version 1.54.0). Two‐sample cis‐MR was conducted using the *TwoSampleMR* package (version 0.6.29). PCA was applied for dimensionality reduction, followed by unsupervised k‐means clustering using the *cluster* package (version 2.1.8.1). Kaplan–Meier survival curves and log‐rank tests were used to compare the clinical outcomes across endotypes. XGBoost‐based prediction models were developed using the *xgboost* package (version 1.7.11.1), with feature selection via LASSO regression, hyperparameter tuning via five‐fold nested cross‐validation with the *nestedcv* package (version 0.8.0), model interpretation via SHAP values generated with the *SHAPforxgboost* package (version 0.1.3), and model performance assessment via AUC.

## 3 Results

### 3.1 Baseline Characteristics

During a median follow‐up of 15 years, 252 of 43,996 participants (0.6%) were newly diagnosed with IPAH. Before matching, incident IPAH cases were older than controls (62.30 vs. 56.71 years, *p*‐value < 0.001), had a greater comorbidity burden, higher baseline BNP (1.54 vs. −0.06, *p*‐value < 0.001) and NT‐proBNP levels (1.85 vs. 0.06, *p*‐value < 0.001), and more frequently reported a slow walking pace. After 1:4 matching, all prespecified baseline characteristics achieved acceptable balance in the incidence cohort (SMDs <0.1), except for heart failure (SMD = 0.121). In the mortality cohort, 60 of 141 participants with IPAH at baseline (42.5%) died during follow‐up. Before matching, deceased participants had a higher proportion of males (56.7% vs. 38.3%, *p*‐value = 0.046), higher baseline BNP (2.12 vs. 1.32, *p*‐value = 0.014) and NT‐proBNP levels (2.74 vs. 1.50, *p*‐value < 0.001), and more frequently reported a slow walking pace (55% vs. 19.8%, *p*‐value < 0.001) (Appendix S5).

### 3.2 Proteins Associated with IPAH Incidence and Mortality

**FIGURE 1:**
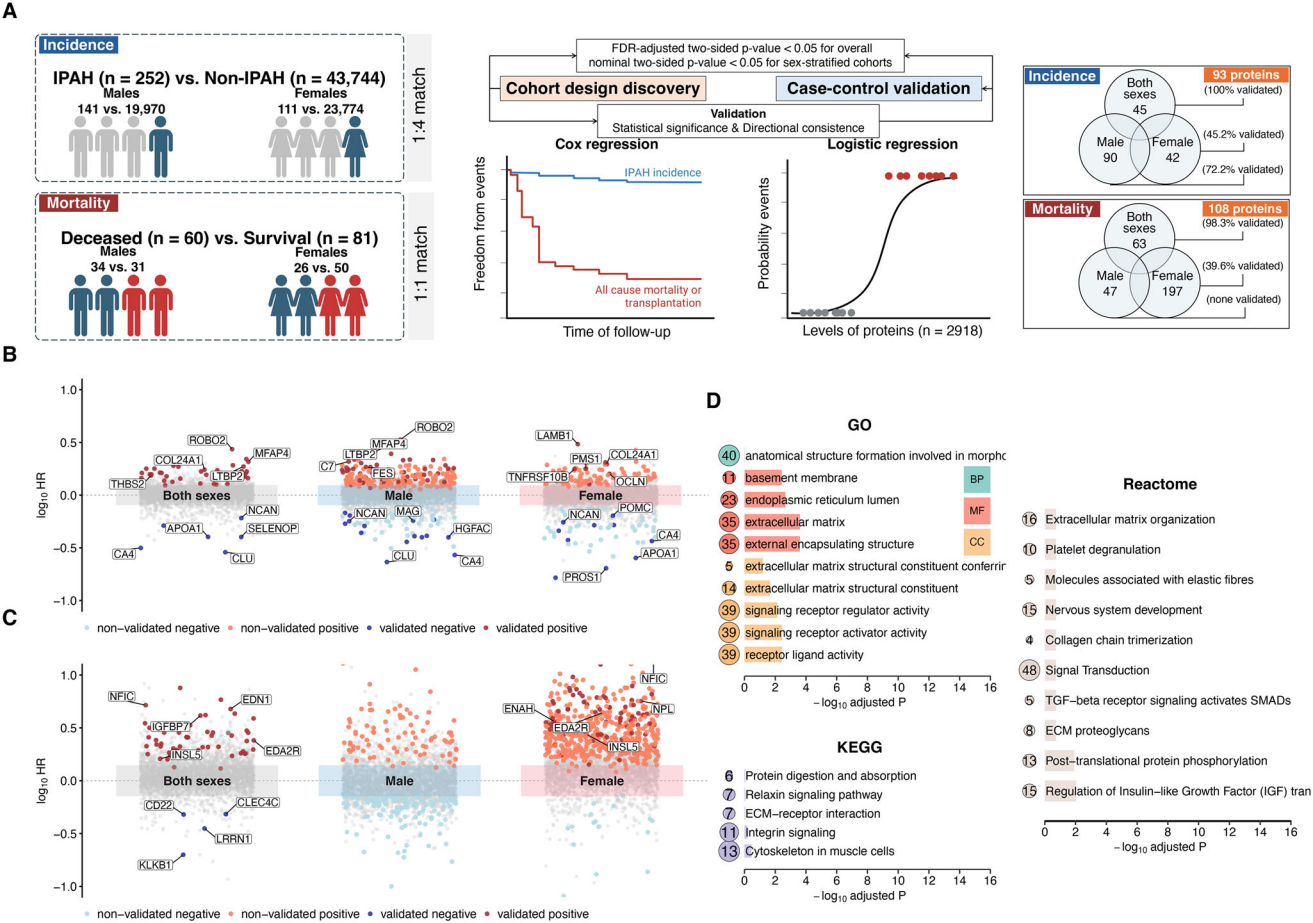
Discovery of proteins associated with IPAH incidence and mortality using epidemiological analyses. Notes: (A) Design and outcome of discovery workflow for proteins associated with IPAH incidence and mortality; Incidence cohort comprised 252 participants who developed IPAH and 43,744 participants who remained free from a recorded IPAH diagnosis; Mortality cohort comprised 60 participants with baseline IPAH who reached the composite endpoint of all‐cause death, lung transplantation, or heart‐lung transplantation, and 81 participants with baseline IPAH who remained alive without transplantation. (B, C) Proteins associated with IPAH incidence (B) and mortality (C) in the combined‐sex and sex‐stratified cohorts. Effect sizes are presented as log_10_(HR) from Cox regression models. Proteins were classified as validated positive or validated negative when they were statistically significant in both analytical designs with concordant directions of effect. Proteins showing significance only in the Cox regression analysis or discordant directions of effect between the two designs were classified as non‐validated positive or non‐validated negative. (D) Enrichment analyses of proteins associated with IPAH incidence and mortality shows the top 10 GO terms, top 5 KEGG pathways, and top 10 Reactome pathways ranked by significance. Abbreviations: IPAH, idiopathic pulmonary arterial hypertension; FDR, false discovery rate; HR, hazard ratio; GO, Gene Ontology; BP, biological process; MF, molecular function; CC, cellular component; KEGG, Kyoto Encyclopedia of genes and genomes.

In the incidence cohort, the primary discovery identified 45 proteins, while supplementary sex‐stratified analyses identified 90 proteins in males and 42 proteins in females after matching on prespecified baseline characteristics and additional adjustment for heart failure. Case‐control validation retained 100% of proteins from the primary analysis, 45.2% from males, and 72.2% from females, resulting in a final incidence‐consensus set of 93 proteins, of which 69 were positively associated and 24 were negatively associated with incident IPAH. In the mortality cohort, discovery yielded 63 mortality‐associated proteins in the primary analysis, 47 in males, and 197 in females after additional adjustment for NT‐proBNP and walking pace. Signals persisted after case‐control validation, with 98.3% of proteins from the primary analysis and 39.6% from the female analysis retained, whereas none of the male‐stratified signals were validated, resulting in a mortality consensus set of 108 proteins. Among these, 104 were positively associated with mortality, and the remaining 4 were negatively associated with mortality. Epidemiologically discovered proteins were mostly enriched in the extracellular matrix and the external encapsulating structure (Figure 1) (Appendix S6).

### 3.3 Genetic Evidence Supported IPAH‐Associated Proteins

**FIGURE 2:**
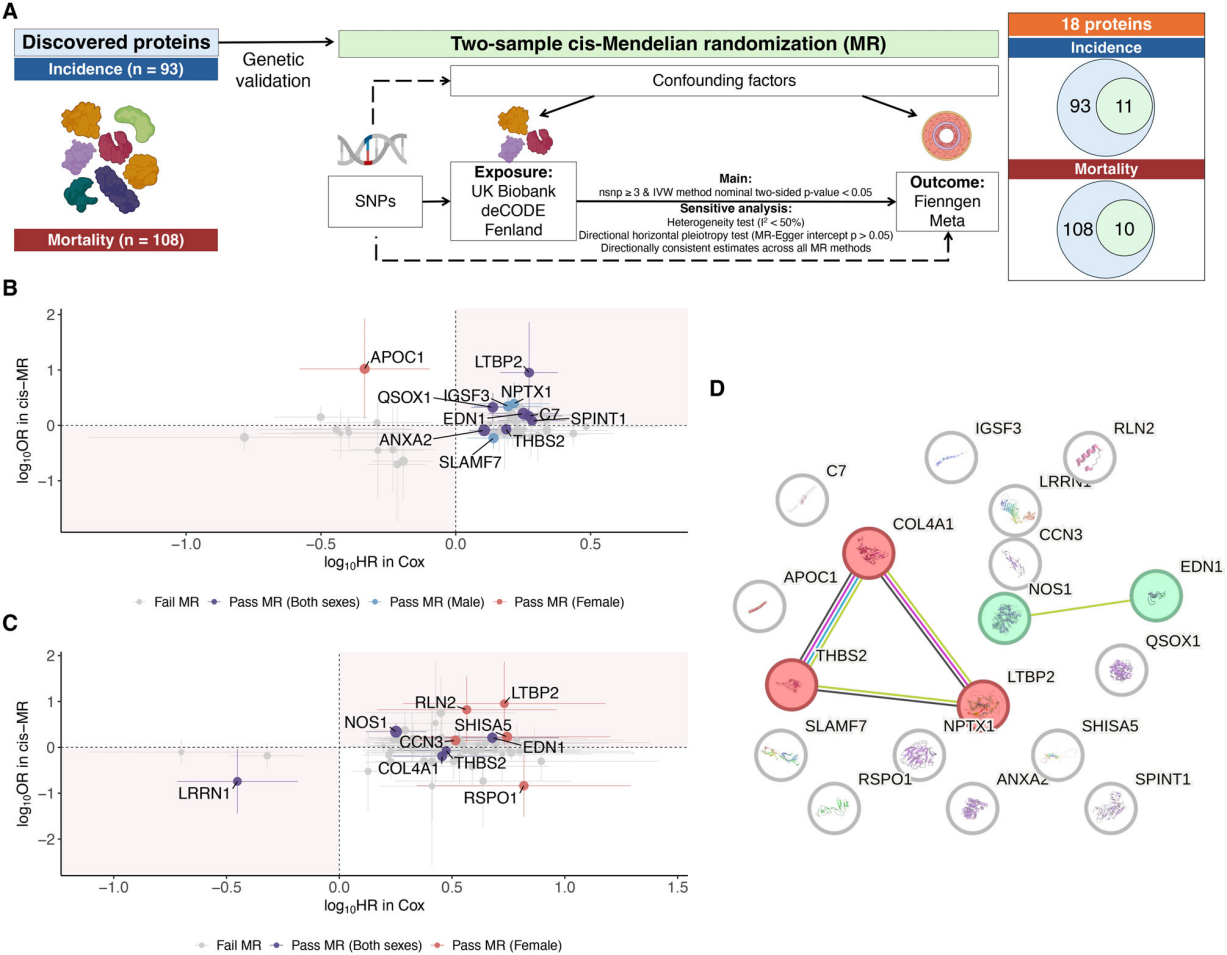
Genetic validation of proteins associated with IPAH incidence and mortality using cis‐MR and sensitivity analyses. Notes: (A) Design and outcome of validation process for proteins associated with IPAH incidence and mortality. In the incidence cohort, 11 of 93 proteins were supported; In the mortality cohort, 10 of 108 proteins were supported. (B, C) Comparison of effect sizes of IPAH incidence (B) and mortality (C) in epidemiological analyses (log_10_HR in Cox regression model, x‐axis) and cis‐MR analyses (log_10_OR in cis‐MR, y‐axis). Proteins in the upper‐right and lower‐left quadrants show concordant directions, with dot colors indicating the sex‐specific source of the proteins identified in the epidemiological discovery analysis. (D) Protein‐protein interaction analysis of genetically validated proteins. Abbreviations: MR, Mendelian randomization; UKB, UK Biobank; IPAH, idiopathic pulmonary arterial hypertension; HR, hazard ratio; OR, odds ratio.

Next, we asked whether these IPAH‐associated proteins were supported by genetic evidence. Among proteins associated with incidence, 11 proteins were confirmed, with seven proteins showing consistently positive associations in both epidemiological and cis‐MR analyses. Notably, LTBP2, QSOX1, EDN1, C7, SPINT1, ANXA2, and THBS2 were identified in the combined‐sex cohort, whereas APOC1, NPTX1, IGSF3, and SLAMF7 were discovered in sex‐stratified cohorts. Of the proteins associated with mortality, 10 in total passed cis‐MR validation, with six showing consistent positive associations and one showing a consistent negative association. Among these proteins, NOS1, COL4A1, THBS2, EDN1, and LRRN1 were identified in combined‐sex cohort, while RLN2, LTBP2, CCN3, SHISA5, and RSPO1 were identified from the female cohort. In total, 18 IPAH‐associated proteins were supported by genetic evidence, and protein–protein interaction analysis revealed two major functional modules: extracellular matrix remodeling and vascular tone regulation (Figure 2) (Appendix S7) (Supplementary Tables).

### 3.4 Cross‐Ancestry and Sensitivity Analysis

Of the 18 proteins supported by genetic evidence, 16 showed cross‐ancestry effect‐direction concordance, and 14 passed the sensitivity analysis, resulting in 12 proteins (i.e., ANXA2, C7, COL4A1, EDN1, IGSF3, LRRN1, LTBP2, NPTX1, QSOX1, SLAMF7, SPINT1, and THBS2) being retained for downstream analyses (Appendices S3 and S4).

### 3.5 Biological Risk Stratification

**FIGURE 3:**
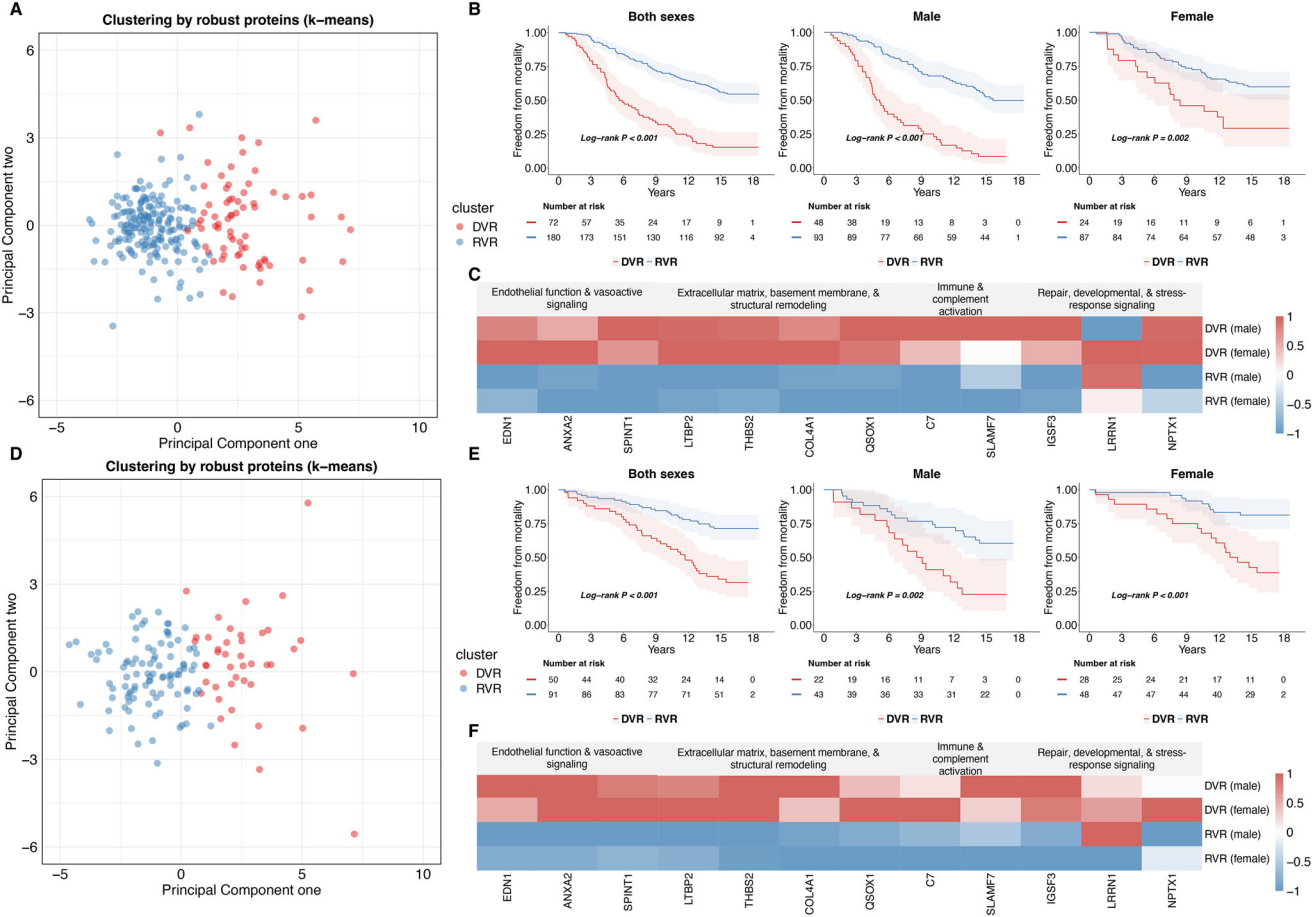
Biological risk stratification for mortality for preclinical and clinical patients with IPAH using clustering analyses. Notes: (A, D) PCA‐based k‐means clustering for preclinical (A) and clinical (D) patients with IPAH. Patients were clustered into two groups termed DVR and RVR. (B, E) Freedom from mortality for preclinical (B) and clinical (E) patients with IPAH grouped by proteomic clusters (i.e., DVR and RVR) and sex (i.e., male and female). (C, F) Sex‐and cluster‐stratified heatmaps of mean protein z‐scores for preclinical (C) and clinical (F) patients with IPAH. Standardized z‐scores were truncated to the range −1 to 1, with higher expression (z closer to 1) shown in red and lower expression (z closer to −1) shown in blue. Abbreviations: IPAH, idiopathic pulmonary arterial hypertension; DVR, dysregulated vascular/ventricular remodeling; RVR, regulated vascular/ventricular remodeling; PCA, principal component analysis.

Proteins confirmed within our analytic framework may serve as promising markers for biological risk stratification. Accordingly, we performed unsupervised clustering, which classified both preclinical and clinical patients into two endotypes. We designated one endotype characterized by globally higher expression of the 12 proteins (i.e., ANXA2, C7, COL4A1, EDN1, IGSF3, LRRN1, LTBP2, NPTX1, QSOX1, SLAMF7, SPINT1, and THBS2) as dysregulated vascular/ventricular remodeling (DVR). In contrast, the other endotype, characterized by relatively lower expression, was designated as regulated vascular/ventricular remodeling (RVR). Notably, patients grouped as DVR had a substantially higher risk of mortality than those grouped as RVR, regardless of sex. Moreover, male patients exhibited a higher mortality risk than females across both endotypes (Figure 3) (Appendix S8).

### 3.6 Prediction Models and Sex‐Differential Protein Contribution

**FIGURE 4:**
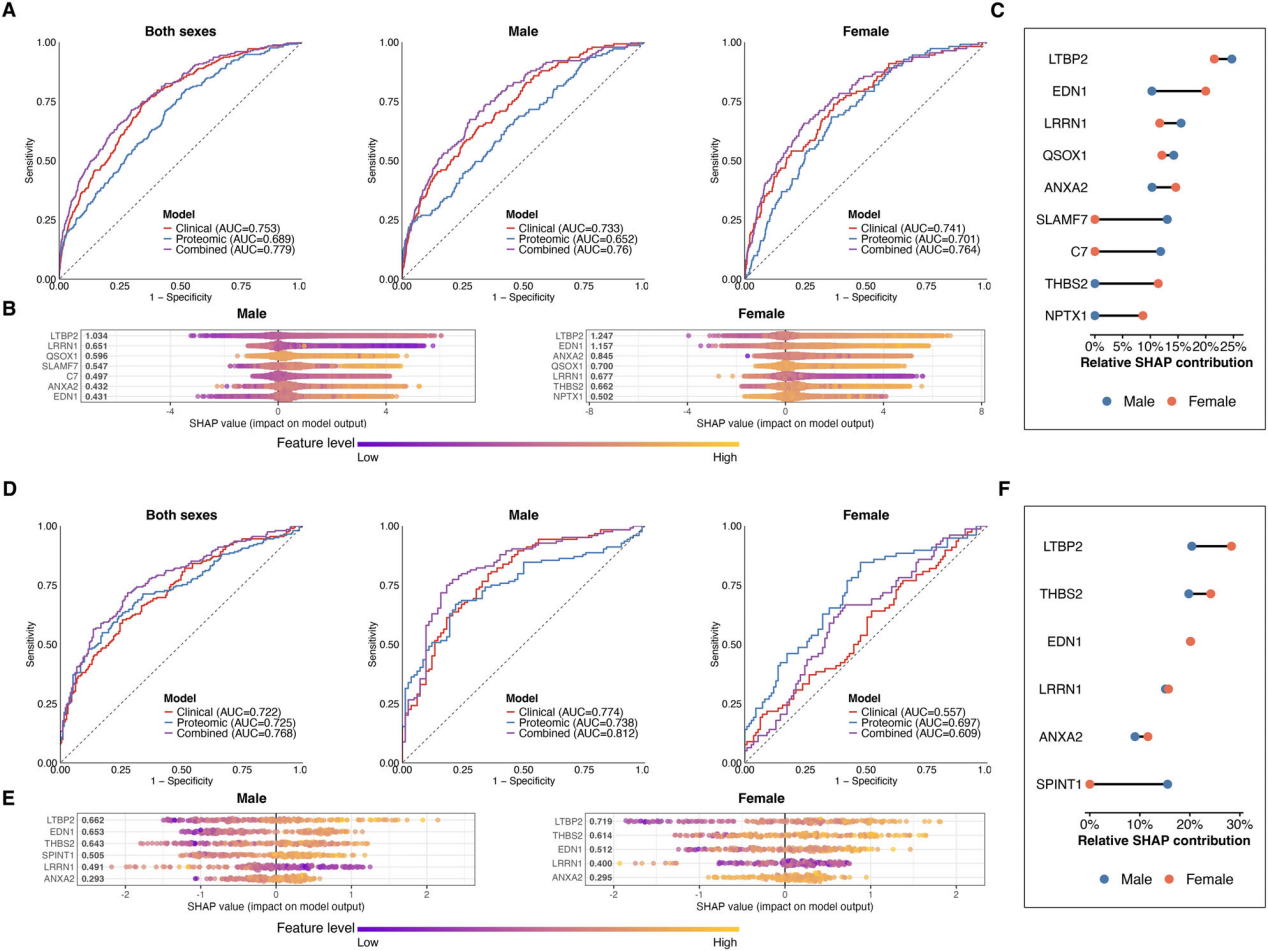
Prediction models for IPAH incidence and mortality integrating biologically plausible proteins. Notes: (A, D) Performance of prediction models for IPAH incidence (A) and mortality (D) in the combined‐sex and sex‐stratified cohorts. (B, E) SHAP‐based ranking of proteins in the proteomic prediction models for IPAH incidence (B) and mortality (E), shown separately for males and females. (C, F) Sex differences in the relative SHAP contributions of predictive proteins to the prediction models for IPAH incidence (C) and mortality (F). Relative SHAP contribution was calculated as the SHAP value of a given protein divided by the sum of SHAP values across all proteins in the corresponding prediction model. Abbreviations: AUC, area under the receiver operating characteristic curve; SHAP, SHapley Additive exPlanations; IPAH, idiopathic pulmonary arterial hypertension.

Given the biologically plausible association of these 12 proteins with disease severity, we further investigated whether they provide independent or incremental predictive value for IPAH onset and prognosis. For incidence prediction, although the proteomic model alone exhibited inferior performance, integrating proteins with clinical predictors improved predictive performance (AUC: 0.779 in both sexes, 0.76 in males, and 0.764 in females). Within these models, LTBP2, LRRN1, QSOX1, and ANXA2 contributed meaningfully to incidence prediction, highlighting their potential utility in early disease detection. Moreover, proteomic predictors also improved predictive performance for mortality, reaching an AUC of 0.768 for both sexes and 0.812 for males, with LTBP2, EDN1, and THBS2 emerging as priority prognostic candidates. The combined mortality model had the lowest Brier score and generally provided the greatest net benefit in decision curve analysis. In the biomarker‐focused analysis, adding the protein panel to BNP/NT‐proBNP increased the mortality AUC from 0.583 to 0.726 (ΔAUC = 0.143, *p*‐value < 0.001) (Figure 4) (Appendix S9).

Notably, sex‐differential patterns in proteomic contributions were observed for IPAH incidence prediction. SLAMF7 and C7 contributed prominently to the male model, whereas EDN1, ANXA2, THBS2, and NPTX1 featured prominently in the female model. Similarly, sex‐differential proteomic patterns were observed in the mortality prediction, with LTBP2 pronounced in females but SPINT1 retained as a predictor of mortality in males (Figure 4).

### 3.7 Therapeutic Targets and Drug Repurposing Opportunities

To assess the therapeutic potential of the 12 high‐confidence IPAH‐associated proteins, we systematically summarized proteomic targets and their related drugs (Table 1). Among all druggable targets, six proteins were linked to existing drugs, two of which have approved IPAH‐targeted therapies. EDN1 is a well‐established pathogenic protein in IPAH, and its receptor antagonist is among the most significant approved targeted therapies. Moreover, although not a direct target, LTBP2 may be biologically linked to *Sotatercept*, as both converge on dysregulated vascular remodeling and TGF‐β‐related signaling in IPAH. In addition to approved therapies for IPAH, other approved drugs identified through the same framework, such as *Fluocinolone acetonide* and *Artenimol* as ANXA2‐linked drugs and *Elotuzumab* as an SLAMF7‐targeting drug, may offer repurposing opportunities.

**TABLE 1:** Integrated prioritization of candidate proteins, druggability, and therapeutic hypotheses for IPAH.

					Therapeutic hypothesis‐generation path ^a^		
Protein	Drug name(s)	Drug groups	Actions	Indications	Drug→protein	Protein→IPAH	Drug→IPAH
**Proteins linked to therapies for IPAH**							
EDN1	Endothelin receptor antagonist	Approved	Receptor inhibitor	PAH	—	+	—
LTBP2 ^b^	Sotatercept	Approved	Upstream signal (TGF‐b) ligand trap	PAH	/	+	—
**Proteins linked to therapies for other disease (for repurposing)**							
ANXA2	Fluocinolone acetonide	Approved	Inducer	Skin conditions	+	+	+
	Artenimol	Approved	Ligand	Plasmodium falciparum	/	+	/
	Lanoteplase	Experimental	Co‐receptor agonist	Myocardial infarction	/	+	/
SLAMF7	Elotuzumab	Approved	Inhibitor	Neoplasm, multiple myeloma	—	+	—
COL4A1	Ocriplasmin	Phase IV	Collagen hydrolytic enzyme	Abnormal retinal morphology	—	+	—
	Collagenase clostridium histolyticum	Phase IV	Collagen hydrolytic enzyme	Dupuytren Contracture	—	+	—
QSOX1	SBI‐183	Experimental	Inhibitor	Tumor growth	—	+	—
**Proteins lacking available targeted agents (for development)**							
C7	/				/	+	/
IGSF3	/				/	+	/
LRRN1	/				/	—	/
NPTX1	/				/	+	/
SPINT1	/				/	+	/
THBS2	/				/	+	/

^a^ In the Drug**→**protein column, “+” indicates that the drug is expected to increase or activate the candidate protein, whereas “‐” indicates inhibition or reduction. In the Protein**→**IPAH column, “+” indicates a potentially disease‐promoting association and “‐” a potentially protective association. The Drug**→**IPAH column indicates the inferred overall therapeutic direction: “‐” suggests a potential reduction in IPAH risk, whereas “+” suggests a potential increase. “/” indicates that no direct or clear directional evidence was available.

^b^ LTBP2 is indirectly linked to *Sotatercept* through the TGF‐β pathway and is not a direct drug target.

## 4 Discussion

Precise classification and targeted therapy are essential for improving prognosis in IPAH. Using large population‐based plasma proteomic data, we identified 12 high‐confidence proteins associated with IPAH. These proteins have translational value in three aspects: first, they define an extremely high‐mortality endotype termed DVR; second, they improve the predictive performance of both incident IPAH and mortality, with sex differences in protein contributions; third, drug‐target repurposing informs future therapeutic development in IPAH.

Progression to RV dysfunction is a key pathophysiological determinant of clinical manifestation and mortality in IPAH [1]. In this context, the 2022 ESC/ERS guidelines included BNP and NT‐proBNP as the only circulating biomarkers incorporated into the risk stratification model, within a broader multiparametric assessment [3]. Our results corroborate this clinical paradigm, demonstrating that LTBP2, another potential RV‐function‐related biomarker, was associated with the risk of incident IPAH and mortality [28]. Moreover, THBS2 has been reported to be associated with maladaptive RV remodeling [29], and our findings further support its contribution to IPAH prognosis. Nevertheless, biomarkers underlying pulmonary vascular pathology remain essential for elucidating disease mechanisms and achieving early detection. EDN1 is a canonical protein upregulated in response to endothelial injury and dysfunction [30], consistent with our observation that higher EDN1 levels were associated with both IPAH onset and mortality. Mechanistic studies have shown that enhanced translation and phosphorylation of ANXA2 promote proliferation and migration of pulmonary artery smooth muscle cells (PASMCs) [31]. Also, reduced endothelial COL4A1 expression may increase susceptibility to vascular injury in IPAH [32]. Corroborating these findings, we found that circulating ANXA2 and COL4A1 were associated with incident IPAH.

In addition to these biomarkers, we further confirmed a panel of biologically plausible, yet relatively underexplored, circulating proteins in IPAH. For example, QSOX1 has been shown to drive vascular smooth muscle cell proliferation and migration during neointima formation in injured arteries [33], a remodeling process that resembles key pathological features of IPAH. Moreover, our findings support C7 as a candidate immune biomarker for the early detection of IPAH, potentially reflecting immunoglobulin‐driven complement activation and its downstream proinflammatory vascular remodeling [34]. Notably, LRRN1, a transmembrane leucine‐rich repeat protein involved in Notch‐associated cell patterning [35], has not, to our knowledge, previously been implicated in IPAH; however, these functions make its association biologically plausible, given the recognized role of JAG1–NOTCH3 signaling in PASMC proliferation [36]. Collectively, within our protein discovery‐validation framework, established biomarkers were recapitulated, while emerging candidates showed biological relevance and mechanistic plausibility.

Prior studies showed that biological stratification based on circulating proteins helps distinguish patients with pulmonary hypertension at increased risk of poor prognosis [13, 15]. J Sweatt et al. identified an immune phenotype characterized by elevated circulating tumor necrosis factor‐related apoptosis‐inducing ligand, C‐C motif chemokine ligand 5 (CCL5), CCL7, CCL4, and macrophage migration inhibitory factor, which was associated with a higher mortality risk among patients with PAH [13]. Also, Boucly et al. applied unsupervised clustering to 156 plasma proteins. They identified a subgroup of pulmonary hypertension patients marked by an upregulated TGF‐β pathway who subsequently experienced poorer clinical outcomes [15]. Extending these observations, our findings suggest another biological endotype, termed DVR‐IPAH, associated with distinctly elevated mortality risk in both sexes. The proteomic signature of this endotype is characterized by enhanced endothelial dysfunction, extracellular matrix remodeling, immune activation, and repair responses, consistent with the clinical observation that pulmonary vascular remodeling and progressive RV failure determine mortality in IPAH. Additionally, emerging data indicate that the male RV is less capable of maintaining adaptive remodeling under chronic pressure overload and is more prone to maladaptive deformation and dysfunction [37]. Based on this, although the proteomic pattern reflecting the DVR‐IPAH endotype appeared largely comparable between sexes, our observational data supported the finding that males nonetheless had a higher risk of death than females.

Our findings indicated that circulating proteins provide independent and incremental prognostic value in IPAH. In previous studies, J Rhodes et al. demonstrated that a panel of nine proteins could identify PAH patients at high risk of mortality without clinical evaluation [12]. At the same time, a minimal six‐protein subset further improved the prediction of transplantation‐free survival when combined with NT‐proBNP [38]. Similarly, the high‐confidence proteins identified in our study provided incremental value beyond BNP and NT‐proBNP. Despite their strong predictive power, the interpretability of predictive parameters derived from traditional observational designs remains a concern. In contrast, although our model showed poorer predictive performance, the proteins selected were replicated by genetic evidence and may therefore have stronger biological support. In addition, the inability to incorporate established clinical risk scores, such as REVEAL, in population‐based cohorts may partly account for the weaker predictive performance of our models. Crucially, however, assessment tools like REVEAL depend heavily on invasive hemodynamics obtained via right heart catheterization, as well as clinician‐dependent subjective assessments, limiting their utility for identifying occult IPAH patients [16]. By achieving acceptable discrimination for incident IPAH using plasma proteins alone, our models demonstrate a distinct translational advantage: they could be used as a noninvasive screening tool suitable for primary care or resource‐limited settings.

Our predictive models also helped understand sex‐differential roles of proteomic biomarkers in IPAH incidence and mortality. Pulmonary vascular‐related proteins, particularly EDN1 and ANXA2, contributed prominently to IPAH incidence in females. Estrogens exert vasculoprotective effects, in part by suppressing EDN1 gene expression and reducing circulating EDN1 [39]. Given the age range of female patients with IPAH in our cohort, many were likely postmenopausal, with reduced estrogen‐mediated vascular protection that may have enhanced the biological effects of EDN1 in IPAH onset. Importantly, hormones are not the sole drivers of sex differences. An in vitro study using shear‐stress‐treated human pulmonary microvascular endothelial cells demonstrated inherent sex divergence in both RNA and protein expression, with a functional manifestation of a higher proliferative rates in male cells under normoxia relative to female cells [40]. Against this background, our findings suggested that such cellular behavior may not directly translate into disease susceptibility, as ANXA2, a protein implicated in PASMC proliferation, contributed more substantially to IPAH incidence in females. Furthermore, as a protein promoting RV fibrosis by stimulating the TGF‐β signalling pathway [28], LTBP2 contributed prominently to mortality prediction in female IPAH patients. By contrast, bone morphogenetic protein (BMP) signaling, as a counter‐regulatory branch of the TGF‐β superfamily, protects the RV against remodeling and dysfunction [41]. A recent genetic epidemiology study identified BMP receptor type 1A (BMPR1A) as a female‐specific genetic determinant of RV phenotype and that was associated with cardiac index in female patients with pulmonary arterial hypertension [42]. Together, these findings provide compelling evidence that BMP/TGF‐β superfamily signaling may have sex‐dependent biological relevance in IPAH. Also, the biological basis of these sex‐related proteins remains complex and warrants further mechanistic validation. Our findings should be interpreted with caution.

Targeted drug treatment has long been the cornerstone of IPAH management, centered on three classic pathways: EDN1 signalling (endothelin‐receptor antagonists), the nitric oxide–cGMP pathway (PDE5 inhibitors and soluble guanylate cyclase stimulators), and the prostacyclin pathway (prostacyclin analogues and IP‐receptor agonists) [3, 4]. Nowadays, agents targeting the BMPR2‐TGF‐β signaling axis (growth differentiation factor modulators) have emerged as a promising fourth therapeutic pathway in IPAH [43]. In our study, two established therapeutic axes were recapitulated. One is EDN1, targeted by endothelin receptor antagonists, and the other is the BMPR2–TGF‐β signalling axis, pharmacologically modulated by *Sotatercept*. Alongside current therapies, our findings highlight *Artenimol* as a potential ANXA2‐linked repurposing candidate for IPAH, as pharmacological inhibition of this pathological mediator has been reported to protect against pulmonary arterial hypertension [31]. In addition, *Artesunate*, a prodrug of *Artenimol* used to treat malaria, is currently being evaluated in an early‐phase clinical trial in patients with pulmonary arterial hypertension (NCT06872112). Overall, the drug repurposing findings of this study provide modest population‐level evidence for further experimental validation of these associated candidates for IPAH.

Several inherent limitations of our study should be acknowledged. First, although a sequential design was used to strengthen internal validity, the lack of independent external cohorts and non‐European ancestry datasets limits the generalizability of our findings to broader populations. Further validation in large, multicenter, clinically well‐characterized IPAH cohorts with diverse ancestral backgrounds is warranted. Second, identifying IPAH cases solely using the ICD‐10 codes in a population‐based cohort, without gold‑standard confirmation by right heart catheterization, may have introduced misclassification. In addition, the volunteer‐based recruitment of UK Biobank may introduce selection bias, as their clinical spectrum may differ from patients treated at specialized IPAH centers. Third, inherent constraints of the UK Biobank dataset limited the availability of several clinical assessments, such as WHO functional class, six‐minute walk distance, direct assessments of hemodynamics, RV function, and detailed information on pulmonary arterial hypertension‐targeted therapies. These unavailable data precluded full adjustment for established clinical predictors, leaving residual confounding possible and restricting assessment of the identified proteins’ incremental predictive value. Therefore, our findings warrant contextualized interpretation and require further validation in disease‐specific cohorts. Fourth, from a translational standpoint, targeted therapies for key candidate proteins remain theoretical possibilities, and the low utilization of existing pathway‐specific agents in the cohort precluded robust pharmacoepidemiologic or real‐world analyses. Fifth, the small sample size and relatively lenient significance threshold in the cross‐ancestry cohort warrant cautious interpretation of these findings. Finally, as systematic genetic evaluation and family‐based assessment were not available, we could not distinguish potentially unrecognized heritable pulmonary arterial hypertension.

In conclusion, we identified 12 biologically plausible proteins associated with IPAH, providing biomarkers for risk stratification, insights into sex‐differential proteins, and candidate therapeutic targets. Using these proteins, we defined a high‐mortality endotype termed DVR and developed early detection and prognostic prediction models for IPAH. Also, we observed sex differences in the proteomic architecture of IPAH and prioritized candidate targets for drug repurposing. Nevertheless, the generalization of our findings should be interpreted with caution, given the absence of independent external validation and the lack of participants with non‐European ancestry backgrounds. Further validation in independent proteomic cohorts, multi‐ancestry populations, and mechanistic studies is warranted to clarify their clinical and therapeutic relevance.

## Author Contributions

**Xinjie Lin**: conceptualization, methodology, software, data curation, investigation, validation, visualization, formal analysis, writing – original draft, writing – review and editing. **Yangchang Zhang**: writing – review and editing, methodology, software, conceptualization. **Yang Pu**: conceptualization, methodology, supervision, resources, writing – review and editing, investigation, writing – original draft. **Duanke Liu**: methodology, software, writing – review and editing, validation. **Tao Lei**: writing – review and editing, data curation, formal analysis. **Kai Ma**: writing – review and editing, supervision. **Qiyu He**: writing – review and editing. **Zheng Dou**: writing – review and editing. **Yuze Liu**: writing – review and editing. **Dengyuan Liu**: writing – review and editing. **Yinge He**: writing – review and editing. **Yanshang Wang**: writing – review and editing. **Xiaojiao Zheng**: writing – review and editing. **Yanbing Ma**: writing – review and editing. **Jianrong Zhou**: writing – review and editing. **Weiding Zhai**: writing – review and editing. **Binbin Su**: resources, supervision, conceptualization, project administration. **Li Chen**: writing – review and editing, conceptualization, methodology, software, validation, investigation, supervision, resources, project administration. **Shoujun Li**: funding acquisition, supervision, conceptualization, project administration.

## Funding

This study was supported by the National Clinical Research Center for Cardiovascular Diseases, Fuwai Hospital, Chinese Academy of Medical Sciences (Grant No. NCRC2024001), National Natural Science Foundation of China (Grant Nos. 82607079 and 82470318), Peking University Medicine Sailing Program for Young Scholar's Scientific & Technological Innovation (Grant No. BMU2026YFJHPY007), Fundamental Research Funds for Central Universities, Chinese Academy of Medical Sciences & Peking Union Medical College (Grant Nos. 3332026116 and 3332026107), Fundamental Research Funds for the Central Universities, Science and Technology Research Program of Chongqing Municpal Education Commission (Grant No. KJQN202415126), NATCM Initiative for Strengthening TCM Evidence‐Based Research, NATCM‐STER, and CSTB2024TIAD‐KPX0032.

## Code Availability Statement

The analysis code used in this study is publicly available on GitHub at https://github.com/drlinxinjie98‐stack/IPAH‐UKBiobank‐proteogenomic‐analysis.

## Conflicts of Interest

The authors declare no conflicts of interest.

## Supporting information




**Supporting File 1**: advs77687‐sup‐0001‐SuppMat.docx.

**Supporting File 2**: advs77687‐sup‐0002‐SuppMat.xlsx.

## Data Availability

Data sharing not applicable to this article as no datasets were generated or analysed during the current study.
