# Supplementary material for: Proteogenomic Profiling of Idiopathic Pulmonary Arterial Hypertension Identifies Sex‐Differential Proteins and Candidate Therapeutic Targets
Source: Adv Sci (Weinh). 2026 Sep 16:e77687. Online ahead of print. doi: 10.1002/advs.77687 (PMC13583112; doi:10.1002/advs.77687)
Supplement: Supplementary file 1 [file ADVS-9999-e77687-s001.docx]

**Appendix**

**Appendix S1. Study flowchart of grouping strategy and analytic workflow**

**Appendix S2. ICD-10 codes of inclusive and exclusive disease**

**Appendix S3. Workflow and outcomes of cross-ancestry effect-direction concordance analysis**

**Appendix S4. Workflow and outcomes of sensitivity analysis**

**Appendix S5. Baseline characteristics of the main cohorts before and after matching**

**Appendix S6. Epidemiological results for proteins significantly associated with IPAH**

**Appendix S7. MR results for proteins epidemiologically associated with IPAH**

**Appendix S8. Detailed information of biological risk stratification**

**Appendix S9. Detailed information of prediction models**

**Appendix S1. Study flowchart of grouping strategy and analytic workflow**

**Supplementary Fig 1.** **Flowchart of discovering IPAH-associated proteins**

**Note: Exclusive criteria for cross-ancestry effect-direction concordance analysis** included missing ethnicity data (n = 264), non-randomized participants (n = 692), post-lung or heart-lung transplantation at baseline (n = 0), and concomitant secondary pulmonary hypertension (n = 17). **Conditions associated with secondary pulmonary hypertension excluded in the sensitivity analysis**, including congenital heart disease, human immunodeficiency virus infection, connective tissue disease, portal hypertension, and hereditary hemorrhagic telangiectasia for Group I (n = 430), left-sided valve disease for Group II (n = 242), chronic obstructive pulmonary disease, interstitial lung disease, and obstructive sleep apnea for Group III (n = 451), and pulmonary embolism for Group IV (n = 150).

**Supplementary Fig 2.** **Flowchart of downstream analyses of discovered IPAH-associated proteins**

**Note:** In the biological risk-stratification, **preclinical IPAH** refers to participants from the incidence cohort who were subsequently diagnosed with IPAH during follow-up, whereas **clinical IPAH** refers to participants with IPAH at baseline in the mortality cohort. Both groups were biologically clustered using the 12 high-confidence proteins to identify endotypes.

**Appendix S2. ICD-10 codes of inclusive and exclusive conditions**

**Inclusive ICD-10 code**

Primary pulmonary hypertension: I27.0

**Exclusive ICD-10 code in the main analysis**

Secondary pulmonary hypertension: I27.2

**Additional exclusive ICD-10 codes in the sensitivity analysis**

1. Pulmonary artery hypertension (Group I)
   a) Congenital heart disease: Q20.0 to Q20.5, Q25.0, Q21.x, Q26.2, and Q26.3
   b) Human immunodeficiency virus infection: B20.x, B21.x, B22.x, B23.x, B24.x, B97.35, Z21.x
   c) Connective tissue disease: L90.0, L94.0, L93.x, M05.x, M06.x, M08.x, M12.0xx, M32.x, M33.x, M34.x, M35.0x, M35.1, M35.8, and M35.9
   d) Portal hypertension: K76.6
   e) Hereditary hemorrhagic telangiectasia: I78.0
2. Pulmonary hypertension due to left heart disease (Group II)
   a) Left-sided valve disease: nonrheumatic of I34.x, I35.x; rheumatic of I05.x, I06.x; multiple valve disease of I08.x; and congenital of Q23.x
3. Pulmonary hypertension due to pulmonary disease (Group III)
   a) Chronic obstructive pulmonary disease: J44.x
   b) Interstitial lung disease: J84.9
   c) Obstructive sleep apnea: G47.3
4. Pulmonary hypertension due to pulmonary embolism (Group IV)
   a) Pulmonary embolism: I26.x

**Appendix S3. Workflow and outcomes of cross-ancestry effect-direction concordance analysis**

**Supplementary Fig 3.** **Detailed exclusive criteria and flowchart of cross-ancestry effect-direction concordance analysis**

**Supplementary Table 1. Cross-ancestry effect-direction concordance analysis (non-European population) of proteins associated with IPAH^1^**

| **Protein**  **name** | **Control**  **(n = 2,548)** | **IPAH**  **(n = 26)** | **OR (95%CI)**  **in the non-European** | **p-value**  **one-sided** | **OR (95%CI)**  **in the European** | **Consistent**  **direction** | **Prioritized** |
| --- | --- | --- | --- | --- | --- | --- | --- |
| ANXA2 | 0.17 (0.70) | 0.34 (0.60) | 1.30 (0.80-2.11) | 0.147 | 1.33 (1.12, 1.57)^2^ | Yes | Yes |
| APOC1 | -0.17 (0.42) | -0.27 (0.32) | 0.79 (0.33-1.87) | 0.297 | 0.42 (0.22, 0.81)^2^ | Yes | No |
| C7 | 0.03 (0.32) | 0.37 (0.50) | 10.26 (4.20-25.09) | <0.001 | 1.98 (1.39, 2.82)^2^ | Yes | Yes |
| CCN3 | -0.10 (0.41) | 0.42 (0.67) | 5.64 (2.99-10.63) | <0.001 | 10.53 (2.17, 51.20)^3^ | Yes | Yes |
| COL4A1 | -0.13 (0.47) | 0.32 (0.57) | 5.67 (2.77-11.62) | <0.001 | 2.98 (1.28, 6.91)^3^ | Yes | Yes |
| EDN1 | -0.09 (0.31) | 0.29 (0.41) | 15.30 (5.99-39.12) | <0.001 | 1.81 (1.28, 2.55)^2^ | Yes | Yes |
| IGSF3 | 0.18 (0.42) | 0.56 (0.55) | 4.73 (2.29-9.76) | <0.001 | 1.62 (1.15, 2.28)^2^ | Yes | Yes |
| LRRN1 | 0.09 (0.46) | -0.16 (0.58) | 0.24 (0.11-0.54) | <0.001 | 0.36 (0.15, 0.89)^3^ | Yes | Yes |
| LTBP2 | -0.11 (0.43) | 0.42 (0.56) | 7.41 (3.68-14.92) | <0.001 | 1.93 (1.45, 2.57)^2^ | Yes | Yes |
| NOS1 | 0.05 (0.68) | 0.48 (0.93) | 1.85 (1.14-3.02) | 0.007 | 2.71 (1.49, 4.94)^3^ | Yes | Yes |
| NPTX1 | -0.01 (0.43) | 0.05 (0.48) | 1.73 (0.69-4.33) | 0.121 | 1.70 (1.20, 2.42)^2^ | Yes | Yes |
| QSOX1 | 0.01 (0.38) | 0.21 (0.31) | 1.62 (1.06-2.47) | 0.013 | 1.51 (1.14, 2.01)^2^ | Yes | Yes |
| RLN2 | 0.32 (1.39) | 0.41 (0.85) | 1.08 (0.83-1.40) | 0.290 | 6.09 (1.58, 23.42)^3^ | Yes | No |
| RSPO1 | -0.16 (0.42) | 0.07 (0.39) | 3.86 (1.77-8.42) | <0.001 | 19.53 (2.85, 133.82)^3^ | Yes | Yes |
| SHISA5 | -0.07 (0.29) | 0.41 (0.65) | 10.64 (5.30-21.34) | <0.001 | 30.21 (3.10, 294.35)^3^ | Yes | Yes |
| SLAMF7 | 0.30 (0.61) | 0.66 (0.75) | 2.07 (1.18-3.63) | 0.006 | 1.46 (1.12, 1.90)^2^ | Yes | Yes |
| SPINT1 | 0.06 (0.29) | 0.20 (0.41) | 2.81 (0.94-8.35) | 0.032 | 2.06 (1.42, 2.99)^2^ | Yes | Yes |
| THBS2 | -0.03 (0.43) | 0.39 (0.70) | 4.42 (2.49-7.83) | <0.001 | 1.63 (1.29, 2.05)^2^ | Yes | Yes |

^1^Logistics regression model adjusted for sex and ethnicity.

^2^ These effect estimates and corresponding 95% confidence intervals were derived from the European IPAH incidence cohort.

^3^ These effect estimates and corresponding 95% confidence intervals were derived from the European mortality cohort.

**Appendix S4. Workflow and outcomes of sensitivity analysis**

**Supplementary Fig 4.** **Detailed exclusive criteria and flowchart of sensitive analysis**

**Supplementary Table 2. Baseline characteristics of sensitivity analysis cohort before and after 1:10 matching**

|  | | **Before matching** | | | **After matching (1:10)** | | | |
| --- | --- | --- | --- | --- | --- | --- | --- | --- |
|  | | **Control (n = 42,626)** | **IPAH (n = 238)** | **p-value** | **Control (n =2,380)** | **IPAH (n = 238)** | **p-value** | **SMD** |
| Age, y | | 56.64 (8.08) | 61.91 (6.36) | <0.001 | 62.12 (6.32) | 61.91 (6.36) | 0.629 | 0.033 |
| Male, % | | 19379 (45.5) | 132 (55.5) | 0.002 | 1333 (56.0) | 132 (55.5) | 0.926 | 0.011 |
| BMI, kg/m^2^ | | 27.32 (4.69) | 29.04 (5.31) | <0.001 | 28.98 (5.47) | 29.04 (5.31) | 0.860 | 0.012 |
| SBP, mmHg | | 137.65 (18.49) | 141.97 (20.55) | <0.001 | 143.10 (20.02) | 141.97 (20.55) | 0.409 | 0.056 |
| DBP, mmHg | | 82.15 (10.08) | 81.24 (10.61) | 0.163 | 81.58 (10.81) | 81.24 (10.61) | 0.640 | 0.032 |
| Smoking, % | | 19499 (45.7) | 128 (53.8) | 0.016 | 1288 (54.1) | 128 (53.8) | 0.975 | 0.007 |
| Drinking, % | | 41320 (96.9) | 222 (93.3) | 0.002 | 2213 (93.0) | 222 (93.3) | 0.971 | 0.012 |
| **Comorbidities** | | | | | | | | |
| Hypertension, % | | 2963 (7.0) | 65 (27.3) | <0.001 | 608 (25.5) | 65 (27.3) | 0.606 | 0.040 |
| CAD, % | | 1142 (2.7) | 36 (15.1) | <0.001 | 333 (14.0) | 36 (15.1) | 0.703 | 0.032 |
| Heart failure, % | | 107 (0.3) | 20 (8.4) | <0.001 | 75 (3.2) | 20 (8.4) | <0.001 | 0.227 |
| AF, % | | 474 (1.1) | 39 (16.4) | <0.001 | 282 (11.8) | 39 (16.4) | 0.053 | 0.131 |
| DM, % | | 722 (1.7) | 25 (10.5) | <0.001 | 191 (8.0) | 25 (10.5) | 0.229 | 0.086 |
| CKD, % | | 44 (0.1) | 3 (1.3) | <0.001 | 26 (1.1) | 3 (1.3) | 1.000 | 0.016 |
| **Lab test** | | | | | | | | |
| ALT, U/L | | 23.40 (14.56) | 22.94 (11.66) | 0.628 | 23.38 (12.47) | 22.94 (11.66) | 0.601 | 0.037 |
| Creatinine umol/L | | 72.14 (18.45) | 83.05 (48.78) | <0.001 | 80.61 (41.64) | 83.05 (48.78) | 0.397 | 0.054 |
| CRP, mg/L | | 2.53 (4.27) | 3.64 (5.89) | <0.001 | 3.47 (5.58) | 3.64 (5.89) | 0.659 | 0.029 |
| Glucose mmol/L | | 5.11 (1.17) | 5.48 (1.48) | <0.001 | 5.49 (1.94) | 5.48 (1.48) | 0.970 | 0.003 |
| HbA1c mmol/mol | | 35.90 (6.45) | 40.02 (10.35) | <0.001 | 39.59 (12.17) | 40.02 (10.35) | 0.603 | 0.038 |
| LDL, mmol/L | | 3.57 (0.87) | 3.22 (0.93) | <0.001 | 3.23 (0.92) | 3.22 (0.93) | 0.825 | 0.015 |
| Triglycerides mmol/L | | 1.74 (1.02) | 1.74 (0.93) | 0.964 | 1.75 (0.95) | 1.74 (0.93) | 0.891 | 0.009 |
| BNP^1^ | | -0.07 (1.44) | 1.47 (2.09) | <0.001 | 1.34 (1.80) | 1.47 (2.09) | 0.298 | 0.066 |
| NT-proBNP^1^ | | 0.05 (1.16) | 1.75 (1.92) | <0.001 | 1.63 (1.58) | 1.75 (1.92) | 0.265 | 0.070 |
| **Exercise capacity** | | | | | | | | |
| Walking pace | |  |  | <0.001 |  |  | 0.846 | 0.039 |
|  | Slow pace | 3006 (7.1) | 58 (24.4) |  | 541 (22.7) | 58 (24.4) |  |  |
|  | Steady average pace | 22451 (52.7) | 133 (55.9) |  | 1363 (57.3) | 133 (55.9) |  |  |
|  | Brisk pace | 17169 (40.3) | 47 (19.7) |  | 476 (20.0) | 47 (19.7) |  |  |
| Total METs per week | | 2626.50 (2614.53) | 2392.32 (2542.45) | 0.168 | 2375.86 (2459.22) | 2392.32 (2542.45) | 0.922 | 0.007 |
| FVC, L | | 3.64 (1.01) | 3.30 (1.01) | <0.001 | 3.32 (0.91) | 3.30 (1.01) | 0.683 | 0.027 |

**Abbreviations:** BMI, body mass index; SBP, systolic blood pressure; DBP, diastolic blood pressure; CAD, coronary artery disease; AF, atrial fibrillation; DM, diabetes mellitus; CKD, chronic kidney disease; ALT, alanine aminotransferase; CRP, C-reactive protein; HbA1c, glycated hemoglobin A1c; LDL, low-density lipoprotein cholesterol; BNP, B-type natriuretic peptide; NT-proBNP, N-terminal pro-B-type natriuretic peptide; METs, metabolic equivalent of tasks; FVC, forced vital capacity; SMD, standardized mean difference.

**Supplementary Table 3. Sensitivity analysis of proteins associated with IPAH**

| **Protein name** | **Both sexes** | | **Male** | | **Female** | |
| --- | --- | --- | --- | --- | --- | --- |
|  | **OR^1^ (95%CI)** | **p-value** | **OR^1^ (95%CI)** | **p-value** | **OR^1^ (95%CI)** | **p-value** |
| ANXA2 | 1.26 (1.07-1.47) | 0.004 | 1.14 (0.92-1.42) | 0.227 | 1.41 (1.10-1.81) | 0.007 |
| APOC1 | 0.65 (0.46-0.92) | 0.015 | 0.68 (0.42-1.10) | 0.114 | 0.48 (0.26-0.88) | 0.018 |
| C7 | 1.96 (1.37-2.81) | <0.001 | 1.98 (1.26-3.12) | 0.003 | 1.85 (1.02-3.36) | 0.042 |
| CCN3 | 1.27 (0.97-1.66) | 0.077 | 1.33 (0.93-1.91) | 0.118 | 1.09 (0.72-1.66) | 0.671 |
| COL4A1 | 1.30 (1.01-1.68) | 0.041 | 1.25 (0.88-1.77) | 0.220 | 1.35 (0.92-1.98) | 0.122 |
| EDN1 | 2.12 (1.49-3.01) | <0.001 | 1.89 (1.19-3.00) | 0.007 | 2.25 (1.28-3.98) | 0.005 |
| IGSF3 | 1.37 (1.06-1.78) | 0.018 | 1.43 (1.02-2.01) | 0.039 | 1.19 (0.77-1.82) | 0.432 |
| LRRN1 | 0.74 (0.55-0.99) | 0.041 | 0.72 (0.49-1.05) | 0.091 | 0.76 (0.48-1.20) | 0.233 |
| LTBP2 | 1.89 (1.42-2.51) | <0.001 | 2.19 (1.49-3.20) | <0.001 | 1.46 (0.93-2.27) | 0.097 |
| NOS1 | 1.16 (0.96-1.42) | 0.130 | 1.14 (0.88-1.49) | 0.317 | 1.16 (0.82-1.63) | 0.404 |
| NPTX1 | 1.51 (1.15-1.98) | 0.003 | 1.55 (1.08-2.20) | 0.016 | 1.45 (0.93-2.26) | 0.099 |
| QSOX1 | 1.37 (1.09-1.72) | 0.008 | 1.50 (1.07-2.09) | 0.018 | 1.17 (0.82-1.66) | 0.381 |
| RLN2 | 1.14 (0.97-1.33) | 0.108 | 1.37 (1.01-1.87) | 0.043 | 1.04 (0.85-1.27) | 0.714 |
| RSPO1 | 1.11 (0.81-1.53) | 0.520 | 1.10 (0.73-1.65) | 0.662 | 1.11 (0.66-1.88) | 0.689 |
| SHISA5 | 1.29 (0.95-1.75) | 0.108 | 1.28 (0.86-1.89) | 0.224 | 1.14 (0.67-1.94) | 0.629 |
| SLAMF7 | 1.17 (0.96-1.42) | 0.125 | 1.31 (1.02-1.69) | 0.037 | 0.95 (0.69-1.31) | 0.754 |
| SPINT1 | 2.10 (1.47-3.01) | <0.001 | 2.26 (1.43-3.57) | <0.001 | 1.84 (1.02-3.34) | 0.044 |
| THBS2 | 1.57 (1.24-1.98) | <0.001 | 1.47 (1.08-1.99) | 0.015 | 1.65 (1.13-2.42) | 0.010 |

^1^Logistics regression model adjusted for heart failure.

**Appendix S5. Baseline characteristics of cohorts before and after matching**

**Supplementary Table 4. Baseline characteristics of IPAH incidence cohort before and after 1:4 matching**

|  | | **Before matching** | | | **After matching (1:4)** | | | |
| --- | --- | --- | --- | --- | --- | --- | --- | --- |
|  | | **Control (n = 43,744)** | **IPAH (n = 252)** | **p-value** | **Control (n = 1,008)** | **IPAH (n = 252)** | **p-value** | **SMD** |
| Age, y | | 56.71 (8.07) | 62.30 (5.93) | <0.001 | 62.62 (5.72) | 62.30 (5.93) | 0.433 | 0.055 |
| Male, % | | 19970 (45.7) | 141 (56.0) | 0.001 | 570 (56.5) | 141 (56.0) | 0.921 | 0.012 |
| BMI, kg/m^2^ | | 27.37 (4.74) | 29.38 (5.76) | <0.001 | 29.66 (6.21) | 29.38 (5.76) | 0.521 | 0.046 |
| SBP, mmHg | | 137.70 (18.52) | 141.66 (20.93) | 0.001 | 142.63 (20.41) | 141.66 (20.93) | 0.504 | 0.047 |
| DBP, mmHg | | 82.14 (10.09) | 81.29 (10.59) | 0.185 | 81.31 (10.98) | 81.29 (10.59) | 0.984 | 0.001 |
| Smoking, % | | 20166 (46.1) | 146 (57.9) | <0.001 | 629 (62.4) | 146 (57.9) | 0.219 | 0.091 |
| Drinking, % | | 42393 (96.9) | 236 (93.7) | 0.005 | 936 (92.9) | 236 (93.7) | 0.761 | 0.032 |
| **Comorbidities** | | | | | | | | |
| Hypertension, % | | 3294 (7.5) | 73 (29.0) | <0.001 | 296 (29.4) | 73 (29.0) | 0.963 | 0.009 |
| CAD, % | | 1335 (3.1) | 38 (15.1) | <0.001 | 152 (15.1) | 38 (15.1) | 1.000 | <0.001 |
| Heart failure, % | | 164 (0.4) | 20 (7.9) | <0.001 | 50 (5.0) | 20 (7.9) | 0.091 | 0.121 |
| AF, % | | 554 (1.3) | 43 (17.1) | <0.001 | 147 (14.6) | 43 (17.1) | 0.376 | 0.068 |
| Left-sided valve disease, % | | 177 (0.4) | 17 (6.7) | <0.001 | 53 (5.3) | 17 (6.7) | 0.442 | 0.063 |
| COPD, % | | 250 (0.6) | 18 (7.1) | <0.001 | 62 (6.2) | 18 (7.1) | 0.665 | 0.040 |
| ILD, % | | 5 (0.0) | 1 (0.4) | 0.012 | 5 (0.5) | 1 (0.4) | 1.000 | 0.015 |
| OSA= 1 (%) | | 196 (0.4) | 3 (1.2) | 0.200 | 11 (1.1) | 3 (1.2) | 1.000 | 0.009 |
| PE, % | | 151 (0.3) | 2 (0.8) | 0.503 | 8 (0.8) | 2 (0.8) | 1.000 | <0.001 |
| DM, % | | 819 (1.9) | 29 (11.5) | <0.001 | 108 (10.7) | 29 (11.5) | 0.803 | 0.025 |
| CKD, % | | 52 (0.1) | 5 (2.0) | <0.001 | 14 (1.4) | 5 (2.0) | 0.686 | 0.046 |
| **Lab test** | | | | | | | | |
| ALT, U/L | | 23.43 (14.56) | 22.57 (11.23) | 0.348 | 22.30 (10.64) | 22.57 (11.23) | 0.725 | 0.024 |
| Creatinine umol/L | | 72.22 (18.59) | 84.30 (54.42) | <0.001 | 82.33 (46.87) | 84.30 (54.42) | 0.563 | 0.039 |
| CRP, mg/L | | 2.58 (4.38) | 4.30 (6.73) | <0.001 | 4.36 (6.85) | 4.30 (6.73) | 0.897 | 0.009 |
| Glucose mmol/L | | 5.11 (1.19) | 5.59 (2.02) | <0.001 | 5.54 (2.09) | 5.59 (2.02) | 0.718 | 0.026 |
| HbA1c mmol/mol | | 35.95 (6.52) | 40.15 (10.94) | <0.001 | 40.47 (13.76) | 40.15 (10.94) | 0.733 | 0.026 |
| LDL, mmol/L | | 3.56 (0.87) | 3.24 (0.96) | <0.001 | 3.21 (0.93) | 3.24 (0.96) | 0.658 | 0.031 |
| Triglycerides mmol/L | | 1.75 (1.02) | 1.76 (0.94) | 0.920 | 1.77 (0.94) | 1.76 (0.94) | 0.814 | 0.017 |
| BNP | | -0.06 (1.45) | 1.54 (2.11) | <0.001 | 1.44 (1.90) | 1.54 (2.11) | 0.464 | 0.050 |
| NT-proBNP | | 0.06 (1.17) | 1.85 (1.97) | <0.001 | 1.75 (1.77) | 1.85 (1.97) | 0.438 | 0.053 |
| **Exercise capacity** | | | | | | | | |
| Walking pace | |  |  | <0.001 |  |  | 0.506 | 0.082 |
|  | Slow pace | 3361 (7.7) | 76 (30.2) |  | 339 (33.6) | 76 (30.2) |  |  |
|  | Steady average pace | 22981 (52.5) | 135 (53.6) |  | 525 (52.1) | 135 (53.6) |  |  |
|  | Brisk pace | 17402 (39.8) | 41 (16.3) |  | 144 (14.3) | 41 (16.3) |  |  |
| Total METs per week | | 2618.28 (2615.18) | 2391.90 (2510.74) | 0.171 | 2406.79 (2637.87) | 2391.90 (2510.74) | 0.936 | 0.006 |
| FVC, L | | 3.63 (1.01) | 3.26 (1.00) | <0.001 | 3.26 (0.94) | 3.26 (1.00) | 0.985 | 0.001 |

**Abbreviations:** IPAH, idiopathic pulmonary arterial hypertension; BMI, body mass index; SBP, systolic blood pressure; DBP, diastolic blood pressure; CAD, coronary artery disease; AF, atrial fibrillation; COPD, chronic obstructive pulmonary disease; ILD, interstitial lung disease; OSA, obstructive sleep apnea; PE, pulmonary embolism; DM, diabetes mellitus; CKD, chronic kidney disease; ALT, alanine aminotransferase; CRP, C-reactive protein; HbA1c, glycated hemoglobin A1c; LDL, low-density lipoprotein cholesterol; BNP, B-type natriuretic peptide; NT-proBNP, N-terminal pro-B-type natriuretic peptide; METs, metabolic equivalent of tasks; FVC, forced vital capacity; SMD, standardized mean difference.

**Supplementary Table 5. Baseline characteristics of IPAH mortality cohort before and after 1:1 matching**

|  | | **Before matching** | | | **After matching (1:1)** | | | |
| --- | --- | --- | --- | --- | --- | --- | --- | --- |
|  | | Survival (n = 81) | Decease (n = 60) | p-value | Survival (n =60) | Deceased (n = 60) | p-value | SMD |
| Age, y | | 60.09 (6.51) | 62.05 (6.70) | 0.082 | 60.63 (6.50) | 62.05 (6.70) | 0.242 | 0.215 |
| Male, % | | 31 (38.3) | 34 (56.7) | 0.046 | 27 (45.0) | 34 (56.7) | 0.273 | 0.235 |
| BMI, kg/m^2^ | | 27.79 (5.13) | 28.60 (5.62) | 0.378 | 28.08 (5.29) | 28.60 (5.62) | 0.600 | 0.096 |
| SBP, mmHg | | 137.98 (17.26) | 135.46 (20.11) | 0.425 | 137.06 (16.69) | 135.46 (20.11) | 0.636 | 0.087 |
| DBP, mmHg | | 80.72 (10.48) | 76.85 (11.07) | 0.036 | 79.58 (10.54) | 76.85 (11.07) | 0.169 | 0.253 |
| Smoking, % | | 41 (50.6) | 37 (61.7) | 0.257 | 31 (51.7) | 37 (61.7) | 0.357 | 0.203 |
| Drinking, % | | 75 (92.6) | 58 (96.7) | 0.506 | 54 (90.0) | 58 (96.7) | 0.272 | 0.270 |
| **Comorbidities** | | | | | | | | |
| Hypertension, % | | 24 (29.6) | 26 (43.3) | 0.133 | 17 (28.3) | 26 (43.3) | 0.128 | 0.317 |
| CAD, % | | 11 (13.6) | 18 (30.0) | 0.030 | 11 (18.3) | 18 (30.0) | 0.201 | 0.275 |
| Heart failure, % | | 17 (21.0) | 26 (43.3) | 0.008 | 15 (25.0) | 26 (43.3) | 0.054 | 0.394 |
| AF, % | | 29 (35.8) | 29 (48.3) | 0.186 | 20 (33.3) | 29 (48.3) | 0.137 | 0.309 |
| Left-sided valve disease, % | | 42 (51.9) | 26 (43.3) | 0.406 | 29 (48.3) | 26 (43.3) | 0.714 | 0.100 |
| COPD, % | | 5 (6.2) | 14 (23.3) | 0.007 | 5 (8.3) | 14 (23.3) | 0.045 | 0.420 |
| ILD, % | | 0 (0.0) | 1 (1.7) | 0.880 | 0 (0.0) | 1 (1.7) | 1.000 | 0.184 |
| OSA= 1 (%) | | 0 (0.0) | 1 (1.7) | 0.880 | 0 (0.0) | 1 (1.7) | 1.000 | 0.184 |
| PE, % | | 5 (6.2) | 4 (6.7) | 1.000 | 4 (6.7) | 4 (6.7) | 1.000 | <0.001 |
| DM, % | | 6 (7.4) | 13 (21.7) | 0.028 | 5 (8.3) | 13 (21.7) | 0.074 | 0.380 |
| CKD, % | | 0 (0.0) | 6 (10.0) | 0.013 | 0 (0.0) | 6 (10.0) | 0.036 | 0.471 |
| **Lab test** | | | | | | | | |
| ALT, U/L | | 23.05 (11.65) | 24.38 (12.00) | 0.508 | 24.37 (12.79) | 24.38 (12.00) | 0.995 | 0.001 |
| Creatinine umol/L | | 73.35 (15.54) | 88.64 (39.66) | 0.002 | 74.43 (16.10) | 88.64 (39.66) | 0.011 | 0.469 |
| CRP, mg/L | | 2.92 (3.49) | 5.17 (9.42) | 0.050 | 3.32 (3.94) | 5.17 (9.42) | 0.163 | 0.256 |
| Glucose mmol/L | | 5.30 (1.73) | 5.80 (2.13) | 0.126 | 5.41 (1.99) | 5.80 (2.13) | 0.304 | 0.189 |
| HbA1c mmol/mol | | 37.55 (7.25) | 40.73 (7.77) | 0.014 | 37.90 (8.04) | 40.73 (7.77) | 0.053 | 0.357 |
| LDL, mmol/L | | 3.34 (0.86) | 2.99 (0.74) | 0.013 | 3.22 (0.82) | 2.99 (0.74) | 0.100 | 0.303 |
| Triglycerides mmol/L | | 1.70 (0.93) | 1.90 (0.98) | 0.210 | 1.80 (1.01) | 1.90 (0.98) | 0.584 | 0.100 |
| BNP | | 1.32 (1.64) | 2.12 (2.22) | 0.014 | 1.35 (1.79) | 2.12 (2.22) | 0.038 | 0.383 |
| NT-proBNP | | 1.50 (1.58) | 2.74 (1.95) | <0.001 | 1.64 (1.61) | 2.74 (1.95) | 0.001 | 0.620 |
| **Exercise capacity** | | | | | | | | |
| Walking pace | |  |  | <0.001 |  |  | 0.005 | 0.625 |
|  | Slow pace | 16 (19.8) | 33 (55.0) |  | 16 (26.7) | 33 (55.0) |  |  |
|  | Steady average pace | 41 (50.6) | 21 (35.0) |  | 30 (50.0) | 21 (35.0) |  |  |
|  | Brisk pace | 24 (29.6) | 6 (10.0) |  | 14 (23.3) | 6 (10.0) |  |  |
| Total METs per week | | 2268.65 (2409.40) | 1842.21 (2198.86) | 0.283 | 2283.64 (2565.27) | 1842.21 (2198.86) | 0.314 | 0.185 |
| FVC, L | | 3.32 (1.07) | 2.93 (1.04) | 0.032 | 3.25 (1.00) | 2.93 (1.04) | 0.097 | 0.305 |

**Abbreviations:** IPAH, idiopathic pulmonary arterial hypertension; BMI, body mass index; SBP, systolic blood pressure; DBP, diastolic blood pressure; CAD, coronary artery disease; AF, atrial fibrillation; COPD, chronic obstructive pulmonary disease; ILD, interstitial lung disease; OSA, obstructive sleep apnea; PE, pulmonary embolism; DM, diabetes mellitus; CKD, chronic kidney disease; ALT, alanine aminotransferase; CRP, C-reactive protein; HbA1c, glycated hemoglobin A1c; LDL, low-density lipoprotein cholesterol; BNP, B-type natriuretic peptide; NT-proBNP, N-terminal pro-B-type natriuretic peptide; METs, metabolic equivalent of tasks; FVC, forced vital capacity; SMD, standardized mean difference.

**Appendix S6. Epidemiological results of proteins significantly associated with IPAH**

**Supplementary Table 6. Epidemiological results of proteins significantly associated with IPAH incidence**

|  | **Cox regression model** | | | **Logistic regression model** | | |  |
| --- | --- | --- | --- | --- | --- | --- | --- |
| **Protein** | **HR (95%CI)** | **p-value (normal)** | **p-value (adjusted)** | **OR (95%CI)** | **p-value (normal)** | **p-value (adjusted)** | **Source** |
| ROBO2 | 2.73 (1.94, 3.84) | < 0.001 | < 0.001 | 2.86 (1.89, 4.33) | < 0.001 | < 0.001 | overall |
| CA4 | 0.32 (0.21, 0.47) | < 0.001 | < 0.001 | 0.28 (0.18, 0.45) | < 0.001 | < 0.001 | overall |
| COL24A1 | 1.74 (1.41, 2.16) | < 0.001 | < 0.001 | 1.73 (1.37, 2.19) | < 0.001 | < 0.001 | overall |
| LTBP2 | 1.87 (1.46, 2.39) | < 0.001 | < 0.001 | 1.93 (1.45, 2.57) | < 0.001 | < 0.001 | overall |
| MFAP4 | 2.07 (1.53, 2.80) | < 0.001 | 0.001 | 2.18 (1.54, 3.09) | < 0.001 | < 0.001 | overall |
| THBS2 | 1.54 (1.29, 1.84) | < 0.001 | 0.001 | 1.63 (1.29, 2.05) | < 0.001 | < 0.001 | overall |
| RBFOX3 | 1.45 (1.23, 1.70) | < 0.001 | 0.003 | 1.59 (1.27, 2.00) | < 0.001 | < 0.001 | overall |
| SPINT1 | 1.92 (1.44, 2.56) | < 0.001 | 0.003 | 2.06 (1.42, 2.99) | < 0.001 | < 0.001 | overall |
| FES | 1.85 (1.41, 2.43) | < 0.001 | 0.006 | 1.75 (1.28, 2.40) | < 0.001 | 0.005 | male |
| PMS1 | 1.79 (1.37, 2.32) | < 0.001 | 0.023 | 1.93 (1.33, 2.78) | < 0.001 | 0.007 | female |
| SETMAR | 1.79 (1.37, 2.33) | < 0.001 | 0.007 | 1.98 (1.41, 2.79) | < 0.001 | < 0.001 | overall |
| AREG | 1.50 (1.24, 1.81) | < 0.001 | 0.007 | 1.54 (1.22, 1.94) | < 0.001 | < 0.001 | overall |
| C7 | 1.86 (1.39, 2.49) | < 0.001 | 0.007 | 1.98 (1.39, 2.82) | < 0.001 | < 0.001 | overall |
| CSRP3 | 1.26 (1.13, 1.41) | < 0.001 | 0.007 | 1.30 (1.14, 1.48) | < 0.001 | < 0.001 | overall |
| MAMDC2 | 1.64 (1.30, 2.06) | < 0.001 | 0.007 | 1.70 (1.28, 2.24) | < 0.001 | < 0.001 | overall |
| WARS | 1.69 (1.32, 2.17) | < 0.001 | 0.008 | 1.77 (1.32, 2.38) | < 0.001 | < 0.001 | overall |
| NCAN | 0.61 (0.48, 0.77) | < 0.001 | 0.011 | 0.59 (0.45, 0.78) | < 0.001 | < 0.001 | overall |
| CEACAM3 | 1.53 (1.24, 1.88) | < 0.001 | 0.011 | 1.58 (1.22, 2.04) | < 0.001 | 0.001 | overall |
| ANGPT2 | 1.52 (1.24, 1.86) | < 0.001 | 0.011 | 1.53 (1.21, 1.94) | < 0.001 | < 0.001 | overall |
| COL5A1 | 1.63 (1.28, 2.07) | < 0.001 | 0.013 | 1.71 (1.28, 2.28) | < 0.001 | < 0.001 | overall |
| TNFRSF10B | 1.58 (1.26, 1.98) | < 0.001 | 0.013 | 1.58 (1.22, 2.06) | < 0.001 | 0.001 | overall |
| EDN1 | 1.78 (1.33, 2.40) | < 0.001 | 0.018 | 1.81 (1.28, 2.55) | < 0.001 | 0.001 | overall |
| GALNT5 | 2.20 (1.47, 3.30) | < 0.001 | 0.018 | 2.26 (1.41, 3.62) | < 0.001 | 0.001 | overall |
| NRP2 | 1.90 (1.36, 2.65) | < 0.001 | 0.022 | 2.04 (1.38, 3.02) | < 0.001 | < 0.001 | overall |
| OCLN | 1.45 (1.20, 1.77) | < 0.001 | 0.022 | 1.47 (1.17, 1.86) | < 0.001 | 0.001 | overall |
| TNNI3 | 1.28 (1.12, 1.45) | < 0.001 | 0.022 | 1.30 (1.12, 1.51) | < 0.001 | 0.001 | overall |
| CD274 | 1.68 (1.28, 2.20) | < 0.001 | 0.022 | 1.71 (1.25, 2.33) | < 0.001 | 0.001 | overall |
| LMNB2 | 1.68 (1.27, 2.21) | < 0.001 | 0.026 | 1.77 (1.26, 2.48) | 0.001 | 0.001 | overall |
| APOA1 | 0.40 (0.25, 0.65) | < 0.001 | 0.026 | 0.37 (0.21, 0.65) | < 0.001 | 0.001 | overall |
| UXS1 | 1.45 (1.19, 1.77) | < 0.001 | 0.026 | 1.52 (1.20, 1.93) | < 0.001 | 0.001 | overall |
| RCOR1 | 1.44 (1.18, 1.75) | < 0.001 | 0.026 | 1.55 (1.20, 2.01) | < 0.001 | 0.001 | overall |
| ANXA2 | 1.27 (1.12, 1.45) | < 0.001 | 0.027 | 1.33 (1.12, 1.57) | 0.001 | 0.001 | overall |
| FGF23 | 1.24 (1.10, 1.39) | < 0.001 | 0.029 | 1.26 (1.09, 1.45) | 0.002 | 0.002 | overall |
| CD80 | 1.65 (1.25, 2.19) | < 0.001 | 0.036 | 1.76 (1.26, 2.45) | < 0.001 | 0.001 | overall |
| MYBPC1 | 1.29 (1.12, 1.48) | < 0.001 | 0.036 | 1.34 (1.14, 1.58) | < 0.001 | 0.001 | overall |
| MAD1L1 | 1.30 (1.12, 1.51) | < 0.001 | 0.036 | 1.37 (1.13, 1.66) | 0.002 | 0.002 | overall |
| VEGFD | 1.61 (1.23, 2.10) | < 0.001 | 0.036 | 1.57 (1.17, 2.11) | 0.003 | 0.003 | overall |
| AOC3 | 1.72 (1.27, 2.33) | < 0.001 | 0.036 | 1.67 (1.19, 2.34) | 0.003 | 0.003 | overall |
| LAMB1 | 3.05 (1.63, 5.69) | < 0.001 | 0.339 | 3.12 (1.53, 6.35) | 0.002 | 0.015 | female |
| CEACAM6 | 1.51 (1.20, 1.90) | < 0.001 | 0.037 | 1.56 (1.19, 2.04) | 0.001 | 0.002 | overall |
| CLU | 0.29 (0.14, 0.58) | < 0.001 | 0.037 | 0.25 (0.11, 0.56) | < 0.001 | 0.001 | overall |
| ADAMTSL2 | 1.56 (1.21, 2.00) | < 0.001 | 0.038 | 1.60 (1.19, 2.15) | 0.002 | 0.002 | overall |
| SELENOP | 0.40 (0.24, 0.67) | < 0.001 | 0.039 | 0.36 (0.20, 0.65) | < 0.001 | 0.001 | overall |
| PTPN9 | 1.61 (1.23, 2.11) | < 0.001 | 0.111 | 1.98 (1.25, 3.15) | 0.004 | 0.01 | male |
| ADGRV1 | 1.39 (1.15, 1.69) | < 0.001 | 0.111 | 1.50 (1.16, 1.96) | 0.002 | 0.009 | male |
| NPL | 1.70 (1.25, 2.29) | < 0.001 | 0.111 | 1.81 (1.27, 2.56) | < 0.001 | 0.008 | male |
| HGFAC | 0.40 (0.23, 0.68) | < 0.001 | 0.111 | 0.36 (0.19, 0.69) | 0.002 | 0.009 | male |
| SFTPA2 | 1.34 (1.13, 1.58) | < 0.001 | 0.047 | 1.33 (1.10, 1.61) | 0.003 | 0.003 | overall |
| SEPTIN8 | 1.58 (1.21, 2.05) | < 0.001 | 0.047 | 1.63 (1.19, 2.25) | 0.002 | 0.003 | overall |
| SRPX | 1.63 (1.23, 2.15) | < 0.001 | 0.047 | 1.68 (1.21, 2.32) | 0.002 | 0.002 | overall |
| ITGBL1 | 1.55 (1.20, 1.99) | < 0.001 | 0.111 | 1.64 (1.20, 2.23) | 0.002 | 0.009 | male |
| PROS1 | 0.51 (0.35, 0.76) | < 0.001 | 0.047 | 0.50 (0.32, 0.78) | 0.002 | 0.002 | overall |
| QSOX1 | 1.37 (1.14, 1.65) | < 0.001 | 0.047 | 1.51 (1.14, 2.01) | 0.004 | 0.004 | overall |
| LYAR | 1.31 (1.12, 1.53) | < 0.001 | 0.111 | 1.47 (1.14, 1.90) | 0.003 | 0.01 | male |
| POMC | 0.64 (0.49, 0.83) | < 0.001 | 0.513 | 0.57 (0.41, 0.80) | < 0.001 | 0.009 | female |
| IGFBP7 | 1.65 (1.23, 2.21) | < 0.001 | 0.111 | 1.72 (1.20, 2.46) | 0.003 | 0.01 | male |
| RFC4 | 2.15 (1.37, 3.40) | < 0.001 | 0.111 | 2.89 (1.43, 5.83) | 0.003 | 0.01 | male |
| SEMA7A | 2.01 (1.33, 3.06) | 0.001 | 0.111 | 2.00 (1.25, 3.21) | 0.004 | 0.01 | male |
| FCRL5 | 1.47 (1.17, 1.86) | 0.001 | 0.111 | 1.52 (1.17, 1.98) | 0.002 | 0.009 | male |
| MAG | 0.57 (0.41, 0.80) | 0.001 | 0.112 | 0.54 (0.36, 0.79) | 0.002 | 0.009 | male |
| CCL19 | 1.17 (1.06, 1.28) | 0.001 | 0.114 | 1.19 (1.06, 1.34) | 0.004 | 0.01 | male |
| NPTX1 | 1.65 (1.21, 2.24) | 0.002 | 0.126 | 1.70 (1.20, 2.42) | 0.003 | 0.01 | male |
| NFASC | 1.82 (1.25, 2.66) | 0.002 | 0.157 | 1.90 (1.22, 2.95) | 0.004 | 0.01 | male |
| ITGB6 | 1.92 (1.26, 2.92) | 0.002 | 0.182 | 1.97 (1.22, 3.19) | 0.006 | 0.011 | male |
| TCOF1 | 1.66 (1.20, 2.31) | 0.002 | 0.182 | 1.92 (1.20, 3.06) | 0.006 | 0.012 | male |
| IGSF3 | 1.56 (1.17, 2.09) | 0.003 | 0.192 | 1.62 (1.15, 2.28) | 0.006 | 0.012 | male |
| ITIH4 | 0.36 (0.18, 0.71) | 0.003 | 0.204 | 0.32 (0.14, 0.69) | 0.004 | 0.01 | male |
| BCAM | 2.18 (1.30, 3.63) | 0.003 | 0.204 | 2.24 (1.24, 4.04) | 0.008 | 0.012 | male |
| KCTD5 | 1.42 (1.12, 1.80) | 0.003 | 0.219 | 1.55 (1.14, 2.12) | 0.005 | 0.011 | male |
| POLR2F | 1.48 (1.14, 1.92) | 0.003 | 0.219 | 1.59 (1.13, 2.23) | 0.008 | 0.012 | male |
| SEZ6L2 | 2.15 (1.29, 3.58) | 0.003 | 0.219 | 2.20 (1.22, 3.95) | 0.008 | 0.012 | male |
| DPY30 | 1.34 (1.10, 1.63) | 0.004 | 0.225 | 1.37 (1.09, 1.73) | 0.008 | 0.012 | male |
| PDXDC1 | 0.44 (0.25, 0.77) | 0.004 | 0.225 | 0.42 (0.23, 0.77) | 0.005 | 0.01 | male |
| BCAN | 0.58 (0.41, 0.84) | 0.004 | 0.225 | 0.55 (0.36, 0.84) | 0.005 | 0.011 | male |
| KLRC1 | 0.52 (0.33, 0.81) | 0.004 | 0.652 | 0.47 (0.28, 0.78) | 0.004 | 0.019 | female |
| ACE2 | 1.37 (1.11, 1.69) | 0.004 | 0.225 | 1.45 (1.13, 1.87) | 0.004 | 0.01 | male |
| FLT3LG | 1.77 (1.20, 2.62) | 0.004 | 0.228 | 1.88 (1.21, 2.92) | 0.005 | 0.011 | male |
| ZBTB17 | 1.33 (1.09, 1.63) | 0.004 | 0.236 | 1.48 (1.10, 1.99) | 0.01 | 0.014 | male |
| TNFSF12 | 0.51 (0.32, 0.81) | 0.004 | 0.236 | 0.46 (0.27, 0.78) | 0.004 | 0.01 | male |
| LY96 | 1.65 (1.17, 2.33) | 0.005 | 0.236 | 1.74 (1.16, 2.60) | 0.007 | 0.012 | male |
| B4GAT1 | 0.37 (0.19, 0.74) | 0.005 | 0.652 | 0.35 (0.16, 0.76) | 0.008 | 0.02 | female |
| SLAMF7 | 1.38 (1.10, 1.73) | 0.005 | 0.249 | 1.46 (1.12, 1.90) | 0.005 | 0.011 | male |
| TMED10 | 0.41 (0.22, 0.77) | 0.005 | 0.249 | 0.38 (0.19, 0.75) | 0.005 | 0.011 | male |
| SLITRK1 | 0.54 (0.35, 0.83) | 0.005 | 0.249 | 0.51 (0.31, 0.84) | 0.007 | 0.012 | male |
| APLP1 | 0.68 (0.52, 0.89) | 0.005 | 0.249 | 0.65 (0.48, 0.89) | 0.007 | 0.012 | male |
| APOC1 | 0.46 (0.26, 0.80) | 0.006 | 0.652 | 0.42 (0.22, 0.81) | 0.01 | 0.02 | female |
| LRTM2 | 0.41 (0.21, 0.78) | 0.006 | 0.288 | 0.37 (0.18, 0.77) | 0.007 | 0.012 | male |
| VNN1 | 1.38 (1.09, 1.75) | 0.007 | 0.297 | 1.43 (1.10, 1.86) | 0.008 | 0.012 | male |
| F2 | 0.16 (0.04, 0.62) | 0.007 | 0.652 | 0.13 (0.03, 0.59) | 0.008 | 0.02 | female |
| APOB | 0.40 (0.20, 0.79) | 0.008 | 0.302 | 0.34 (0.16, 0.76) | 0.008 | 0.012 | male |
| PRKCQ | 0.52 (0.32, 0.84) | 0.008 | 0.652 | 0.49 (0.28, 0.83) | 0.008 | 0.02 | female |
| EPB41L5 | 0.64 (0.46, 0.89) | 0.009 | 0.302 | 0.60 (0.41, 0.88) | 0.008 | 0.012 | male |
| CLEC4M | 1.61 (1.13, 2.29) | 0.009 | 0.302 | 1.71 (1.15, 2.54) | 0.008 | 0.012 | male |

**Abbreviations:** IPAH, idiopathic pulmonary arterial hypertension.

**Supplementary Table 7. Epidemiological results of proteins significantly associated with mortality**

|  | **Cox regression model** | | | **Logistic regression model** | | |  |
| --- | --- | --- | --- | --- | --- | --- | --- |
| **Protein** | **HR (95%CI)** | **p-value (normal)** | **p-value (adjusted)** | **OR (95%CI)** | **p-value (normal)** | **p-value (adjusted)** | **Source** |
| NFIC | 5.19 (2.68, 10.03) | < 0.001 | 0.003 | 12.02 (2.80, 51.71) | < 0.001 | 0.004 | overall |
| INSL5 | 1.61 (1.31, 1.99) | < 0.001 | 0.004 | 2.57 (1.59, 4.17) | < 0.001 | 0.004 | overall |
| EDN1 | 4.77 (2.42, 9.42) | < 0.001 | 0.004 | 14.44 (3.19, 65.38) | < 0.001 | 0.004 | overall |
| IGFBP7 | 4.14 (2.22, 7.71) | < 0.001 | 0.004 | 5.51 (1.64, 18.56) | 0.006 | 0.008 | overall |
| EDA2R | 2.41 (1.64, 3.55) | < 0.001 | 0.004 | 5.44 (2.25, 13.15) | < 0.001 | 0.004 | overall |
| HGF | 3.50 (2.00, 6.13) | < 0.001 | 0.005 | 7.26 (2.40, 21.97) | < 0.001 | 0.004 | overall |
| FABP3 | 1.82 (1.39, 2.39) | < 0.001 | 0.005 | 4.17 (1.86, 9.35) | < 0.001 | 0.004 | overall |
| NPL | 2.86 (1.74, 4.68) | < 0.001 | 0.008 | 4.62 (1.91, 11.17) | < 0.001 | 0.004 | overall |
| NPTX2 | 4.31 (2.16, 8.62) | < 0.001 | 0.008 | 5.24 (1.66, 16.52) | 0.005 | 0.008 | overall |
| THBS2 | 2.98 (1.78, 5.00) | < 0.001 | 0.008 | 2.93 (1.18, 7.26) | 0.02 | 0.023 | overall |
| SEPTIN8 | 3.34 (1.88, 5.93) | < 0.001 | 0.008 | 6.26 (1.93, 20.34) | 0.002 | 0.005 | overall |
| COL4A1 | 2.86 (1.73, 4.73) | < 0.001 | 0.009 | 2.98 (1.28, 6.91) | 0.011 | 0.014 | overall |
| TIMP1 | 4.18 (2.10, 8.30) | < 0.001 | 0.009 | 9.58 (2.39, 38.48) | 0.001 | 0.004 | overall |
| RNASE6 | 1.89 (1.38, 2.60) | < 0.001 | 0.011 | 8.26 (2.24, 30.49) | 0.002 | 0.004 | overall |
| BAMBI | 3.16 (1.79, 5.58) | < 0.001 | 0.011 | 5.67 (1.65, 19.52) | 0.006 | 0.008 | overall |
| RBFOX3 | 2.24 (1.50, 3.33) | < 0.001 | 0.011 | 4.48 (1.77, 11.33) | 0.002 | 0.004 | overall |
| CCL21 | 4.78 (2.20, 10.42) | < 0.001 | 0.021 | 7.72 (1.78, 33.47) | 0.006 | 0.023 | female |
| GDF15 | 1.69 (1.30, 2.20) | < 0.001 | 0.012 | 6.62 (2.73, 16.04) | < 0.001 | 0.002 | overall |
| KIF1C | 6.72 (2.60, 17.41) | < 0.001 | 0.021 | 22.16 (2.18, 225.41) | 0.009 | 0.023 | female |
| CCN3 | 3.28 (1.80, 5.95) | < 0.001 | 0.022 | 10.53 (2.17, 51.20) | 0.004 | 0.023 | female |
| LTBP3 | 1.66 (1.28, 2.14) | < 0.001 | 0.016 | 4.35 (1.92, 9.85) | < 0.001 | 0.004 | overall |
| FLT1 | 7.55 (2.70, 21.11) | < 0.001 | 0.016 | 15.81 (2.45, 102.02) | 0.004 | 0.007 | overall |
| ACE2 | 2.08 (1.43, 3.03) | < 0.001 | 0.016 | 3.32 (1.61, 6.87) | 0.001 | 0.004 | overall |
| NT5C1A | 2.67 (1.61, 4.43) | < 0.001 | 0.017 | 10.66 (2.90, 39.13) | < 0.001 | 0.004 | overall |
| CD22 | 0.48 (0.33, 0.70) | < 0.001 | 0.017 | 0.27 (0.12, 0.64) | 0.003 | 0.006 | overall |
| GCHFR | 2.59 (1.58, 4.23) | < 0.001 | 0.017 | 4.50 (1.74, 11.67) | 0.002 | 0.005 | overall |
| CHCHD6 | 5.84 (2.34, 14.61) | < 0.001 | 0.018 | 23.32 (3.63, 149.72) | < 0.001 | 0.004 | overall |
| TMED4 | 14.46 (3.60, 58.01) | < 0.001 | 0.026 | 24.30 (2.27, 260.12) | 0.008 | 0.023 | female |
| COL6A3 | 3.61 (1.85, 7.04) | < 0.001 | 0.026 | 9.19 (2.45, 34.55) | 0.001 | 0.023 | female |
| SEL1L | 2.11 (1.43, 3.12) | < 0.001 | 0.019 | 6.15 (1.44, 26.35) | 0.014 | 0.017 | overall |
| TST | 2.58 (1.57, 4.24) | < 0.001 | 0.026 | 3.45 (1.44, 8.27) | 0.005 | 0.023 | female |
| GGT1 | 1.95 (1.37, 2.80) | < 0.001 | 0.024 | 2.79 (1.41, 5.48) | 0.003 | 0.006 | overall |
| NOS1 | 1.79 (1.31, 2.43) | < 0.001 | 0.024 | 2.71 (1.49, 4.94) | 0.001 | 0.004 | overall |
| COX6B1 | 3.30 (1.74, 6.25) | < 0.001 | 0.024 | 8.40 (1.86, 38.01) | 0.006 | 0.008 | overall |
| CDHR2 | 1.59 (1.24, 2.05) | < 0.001 | 0.025 | 1.91 (1.25, 2.93) | 0.003 | 0.006 | overall |
| HRC | 2.67 (1.57, 4.55) | < 0.001 | 0.025 | 5.23 (1.44, 19.02) | 0.012 | 0.014 | overall |
| LMOD1 | 2.37 (1.49, 3.79) | < 0.001 | 0.025 | 4.17 (1.45, 11.95) | 0.008 | 0.011 | overall |
| GIPC2 | 2.85 (1.61, 5.02) | < 0.001 | 0.025 | 5.43 (1.66, 17.83) | 0.005 | 0.008 | overall |
| ADAM12 | 5.28 (2.14, 13.02) | < 0.001 | 0.035 | 9.47 (2.08, 43.02) | 0.004 | 0.023 | female |
| HSPB6 | 2.02 (1.38, 2.96) | < 0.001 | 0.025 | 4.06 (1.75, 9.43) | 0.001 | 0.004 | overall |
| KLK4 | 1.70 (1.27, 2.27) | < 0.001 | 0.028 | 1.72 (1.06, 2.79) | 0.027 | 0.03 | overall |
| EFHD1 | 2.58 (1.53, 4.35) | < 0.001 | 0.031 | 5.68 (1.86, 17.29) | 0.002 | 0.005 | overall |
| ARNT | 6.30 (2.27, 17.45) | < 0.001 | 0.039 | 11.80 (1.97, 70.71) | 0.007 | 0.023 | female |
| TNFRSF19 | 5.66 (2.16, 14.78) | < 0.001 | 0.039 | 20.28 (3.33, 123.63) | 0.001 | 0.023 | female |
| IL17C | 1.73 (1.28, 2.35) | < 0.001 | 0.039 | 2.55 (1.28, 5.09) | 0.008 | 0.023 | female |
| ACVRL1 | 4.91 (2.03, 11.90) | < 0.001 | 0.039 | 44.57 (4.08, 486.78) | 0.002 | 0.023 | female |
| IL36G | 1.88 (1.32, 2.67) | < 0.001 | 0.032 | 3.07 (1.52, 6.21) | 0.002 | 0.005 | overall |
| CLEC4C | 0.48 (0.32, 0.72) | < 0.001 | 0.032 | 0.31 (0.14, 0.69) | 0.004 | 0.007 | overall |
| ANGPT2 | 3.41 (1.72, 6.77) | < 0.001 | 0.039 | 4.09 (1.47, 11.42) | 0.007 | 0.023 | female |
| DTNB | 1.97 (1.35, 2.88) | < 0.001 | 0.032 | 4.43 (1.72, 11.41) | 0.002 | 0.005 | overall |
| TNFRSF10B | 2.56 (1.51, 4.35) | < 0.001 | 0.032 | 5.65 (2.08, 15.35) | < 0.001 | 0.004 | overall |
| PGF | 2.57 (1.52, 4.37) | < 0.001 | 0.032 | 5.15 (1.65, 16.12) | 0.005 | 0.008 | overall |
| KLK3 | 1.31 (1.12, 1.52) | < 0.001 | 0.036 | 1.40 (1.09, 1.81) | 0.009 | 0.011 | overall |
| RBP5 | 1.72 (1.26, 2.34) | < 0.001 | 0.036 | 2.58 (1.42, 4.70) | 0.002 | 0.005 | overall |
| FABP5 | 1.93 (1.33, 2.82) | < 0.001 | 0.046 | 2.99 (1.33, 6.73) | 0.008 | 0.023 | female |
| FDX2 | 2.20 (1.40, 3.46) | < 0.001 | 0.04 | 3.52 (1.45, 8.59) | 0.006 | 0.008 | overall |
| MB | 1.74 (1.26, 2.39) | < 0.001 | 0.04 | 2.79 (1.50, 5.18) | 0.001 | 0.004 | overall |
| RSPO1 | 6.58 (2.21, 19.63) | < 0.001 | 0.051 | 19.53 (2.85, 133.82) | 0.002 | 0.023 | female |
| GM2A | 2.07 (1.36, 3.16) | < 0.001 | 0.044 | 3.20 (1.20, 8.55) | 0.02 | 0.023 | overall |
| KLKB1 | 0.20 (0.08, 0.51) | < 0.001 | 0.044 | 0.11 (0.02, 0.53) | 0.005 | 0.008 | overall |
| REN | 1.34 (1.13, 1.59) | < 0.001 | 0.044 | 1.86 (1.32, 2.62) | < 0.001 | 0.004 | overall |
| SPINK8 | 3.89 (1.76, 8.58) | < 0.001 | 0.045 | 6.81 (1.53, 30.34) | 0.012 | 0.014 | overall |
| LAMA4 | 7.86 (2.35, 26.25) | < 0.001 | 0.052 | 17.51 (2.83, 108.39) | 0.002 | 0.023 | female |
| GAS2 | 2.90 (1.56, 5.40) | < 0.001 | 0.045 | 5.95 (1.98, 17.88) | 0.002 | 0.004 | overall |
| FKBP4 | 2.85 (1.54, 5.28) | < 0.001 | 0.045 | 4.28 (1.40, 13.07) | 0.011 | 0.014 | overall |
| PSMG3 | 1.84 (1.29, 2.64) | < 0.001 | 0.045 | 2.40 (1.16, 4.95) | 0.018 | 0.021 | overall |
| CHCHD10 | 1.94 (1.31, 2.87) | < 0.001 | 0.046 | 3.73 (1.57, 8.88) | 0.003 | 0.006 | overall |
| CPXM2 | 7.34 (2.26, 23.81) | < 0.001 | 0.053 | 20.47 (2.22, 188.97) | 0.008 | 0.023 | female |
| PALM2 | 2.12 (1.36, 3.31) | < 0.001 | 0.047 | 4.55 (1.74, 11.90) | 0.002 | 0.005 | overall |
| ENAH | 2.10 (1.35, 3.25) | < 0.001 | 0.047 | 3.42 (1.44, 8.10) | 0.005 | 0.008 | overall |
| WFDC2 | 1.80 (1.27, 2.55) | < 0.001 | 0.047 | 3.74 (1.73, 8.09) | < 0.001 | 0.004 | overall |
| LRRN1 | 0.35 (0.19, 0.66) | < 0.001 | 0.047 | 0.36 (0.15, 0.89) | 0.026 | 0.029 | overall |
| NEB | 2.42 (1.43, 4.10) | 0.001 | 0.048 | 6.27 (2.04, 19.28) | 0.001 | 0.004 | overall |
| FGF5 | 4.95 (1.91, 12.83) | 0.001 | 0.053 | 9.43 (1.92, 46.31) | 0.006 | 0.023 | female |
| GRP | 2.12 (1.35, 3.33) | 0.001 | 0.053 | 5.10 (1.67, 15.54) | 0.004 | 0.023 | female |
| MDK | 2.10 (1.35, 3.28) | 0.001 | 0.049 | 2.82 (1.34, 5.95) | 0.006 | 0.009 | overall |
| COLEC12 | 6.13 (2.05, 18.36) | 0.001 | 0.056 | 14.60 (2.40, 88.91) | 0.004 | 0.023 | female |
| LTBP2 | 5.39 (1.92, 15.13) | 0.001 | 0.056 | 9.08 (1.78, 46.17) | 0.008 | 0.023 | female |
| SHISA5 | 5.55 (1.94, 15.85) | 0.001 | 0.056 | 30.21 (3.10, 294.35) | 0.003 | 0.023 | female |
| TSPYL1 | 6.09 (2.01, 18.41) | 0.001 | 0.056 | 9.95 (1.78, 55.60) | 0.009 | 0.023 | female |
| EFNA4 | 4.34 (1.76, 10.70) | 0.001 | 0.056 | 18.90 (3.03, 117.81) | 0.002 | 0.023 | female |
| TGFB1 | 6.34 (2.04, 19.70) | 0.001 | 0.056 | 10.89 (1.99, 59.49) | 0.006 | 0.023 | female |
| MMP7 | 3.58 (1.63, 7.87) | 0.002 | 0.057 | 6.93 (1.75, 27.50) | 0.006 | 0.023 | female |
| LEFTY2 | 3.37 (1.58, 7.18) | 0.002 | 0.057 | 4.85 (1.56, 15.07) | 0.006 | 0.023 | female |
| IGFBP4 | 2.46 (1.39, 4.36) | 0.002 | 0.067 | 5.28 (1.80, 15.51) | 0.002 | 0.023 | female |
| NUCB2 | 4.73 (1.76, 12.71) | 0.002 | 0.068 | 13.49 (2.25, 80.82) | 0.004 | 0.023 | female |
| PENK | 4.29 (1.66, 11.08) | 0.003 | 0.079 | 34.86 (3.99, 304.54) | 0.001 | 0.023 | female |
| LAYN | 4.02 (1.60, 10.10) | 0.003 | 0.085 | 9.83 (1.96, 49.29) | 0.005 | 0.023 | female |
| ERP44 | 12.59 (2.35, 67.45) | 0.003 | 0.085 | 40.15 (2.55, 631.98) | 0.009 | 0.023 | female |
| SPON2 | 4.44 (1.65, 11.97) | 0.003 | 0.086 | 9.42 (1.85, 48.04) | 0.007 | 0.023 | female |
| HDGF | 2.14 (1.29, 3.54) | 0.003 | 0.086 | 2.87 (1.30, 6.34) | 0.009 | 0.023 | female |
| OGN | 3.27 (1.48, 7.21) | 0.003 | 0.086 | 5.88 (1.79, 19.29) | 0.003 | 0.023 | female |
| IL17RA | 3.85 (1.55, 9.53) | 0.004 | 0.091 | 9.45 (2.31, 38.73) | 0.002 | 0.023 | female |
| HSPG2 | 3.21 (1.45, 7.13) | 0.004 | 0.094 | 11.72 (2.03, 67.74) | 0.006 | 0.023 | female |
| SCRG1 | 4.20 (1.55, 11.42) | 0.005 | 0.109 | 10.63 (1.95, 58.06) | 0.006 | 0.023 | female |
| LGALS1 | 3.56 (1.47, 8.63) | 0.005 | 0.111 | 8.79 (1.92, 40.23) | 0.005 | 0.023 | female |
| RLN2 | 3.67 (1.47, 9.19) | 0.005 | 0.117 | 6.09 (1.58, 23.42) | 0.009 | 0.023 | female |
| CALCB | 3.35 (1.43, 7.88) | 0.006 | 0.117 | 7.32 (1.69, 31.73) | 0.008 | 0.023 | female |
| TAFA5 | 3.47 (1.44, 8.41) | 0.006 | 0.119 | 6.15 (1.68, 22.59) | 0.006 | 0.023 | female |
| ASGR1 | 3.36 (1.42, 7.97) | 0.006 | 0.12 | 8.89 (1.95, 40.49) | 0.005 | 0.023 | female |
| COL18A1 | 4.99 (1.59, 15.67) | 0.006 | 0.12 | 13.11 (2.07, 83.20) | 0.006 | 0.023 | female |
| TNFRSF1A | 3.06 (1.38, 6.80) | 0.006 | 0.121 | 6.94 (1.64, 29.41) | 0.009 | 0.023 | female |
| CKAP4 | 5.79 (1.65, 20.35) | 0.006 | 0.121 | 12.59 (1.86, 85.13) | 0.009 | 0.024 | female |
| STC1 | 3.11 (1.36, 7.13) | 0.007 | 0.131 | 8.65 (1.84, 40.66) | 0.006 | 0.023 | female |
| TNFRSF12A | 2.82 (1.31, 6.05) | 0.008 | 0.133 | 6.76 (1.76, 25.96) | 0.005 | 0.023 | female |
| PIGR | 2.74 (1.30, 5.74) | 0.008 | 0.133 | 9.52 (1.93, 46.95) | 0.006 | 0.023 | female |
| FABP1 | 1.57 (1.12, 2.20) | 0.009 | 0.137 | 2.26 (1.25, 4.08) | 0.007 | 0.023 | female |
| COL5A1 | 4.01 (1.40, 11.49) | 0.01 | 0.144 | 10.30 (1.79, 59.17) | 0.009 | 0.023 | female |

**Abbreviations:** IPAH, idiopathic pulmonary arterial hypertension.

**Supplementary Table 8. Associations of the 12 identified proteins with incident IPAH using Cox regression after the 1:4 matching (non-imputed proteomic data)**

| **Protein** | **IPAH cases with available proteomic data, n** | **HR (95%CI)** | **p-value** |
| --- | --- | --- | --- |
| EDN1 | 234 | 1.73 (1.28, 2.33) | < 0.001 |
| ANXA2 | 231 | 1.30 (1.13, 1.50) | < 0.001 |
| SPINT1 | 246 | 2.01 (1.51, 2.68) | < 0.001 |
| LTBP2 | 248 | 1.89 (1.48, 2.41) | < 0.001 |
| THBS2 | 246 | 1.58 (1.33, 1.89) | < 0.001 |
| COL4A1 | 246 | 1.37 (1.09, 1.72) | 0.006 |
| QSOX1 | 229 | 1.35 (1.13, 1.63) | 0.001 |
| C7 | 226 | 1.86 (1.39, 2.50) | < 0.001 |
| SLAMF7 | 243 | 1.16 (0.97, 1.38) | 0.1 |
| IGSF3 | 244 | 1.39 (1.10, 1.77) | 0.006 |
| LRRN1 | 244 | 0.83 (0.63, 1.08) | 0.158 |
| NPTX1 | 246 | 1.32 (1.04, 1.68) | 0.025 |

**Abbreviations:** IPAH, idiopathic pulmonary arterial hypertension.

**Supplementary Table 9. Associations of the 12 identified proteins with mortality using Cox regression after the 1:1 matching (non-imputed proteomic data)**

| **Protein** | **IPAH cases with available proteomic data, n** | **HR (95%CI)** | **p-value** |
| --- | --- | --- | --- |
| EDN1 | 55 | 5.49 (2.60, 11.58) | < 0.001 |
| ANXA2 | 55 | 1.04 (0.66, 1.63) | 0.869 |
| SPINT1 | 59 | 1.15 (0.44, 2.98) | 0.78 |
| LTBP2 | 59 | 3.15 (1.49, 6.63) | 0.003 |
| THBS2 | 59 | 3.12 (1.84, 5.30) | < 0.001 |
| COL4A1 | 59 | 2.95 (1.77, 4.91) | < 0.001 |
| QSOX1 | 53 | 2.19 (1.26, 3.80) | 0.006 |
| C7 | 52 | 3.68 (1.56, 8.72) | 0.003 |
| SLAMF7 | 60 | 1.06 (0.68, 1.65) | 0.81 |
| IGSF3 | 58 | 1.52 (0.80, 2.88) | 0.198 |
| LRRN1 | 58 | 0.36 (0.19, 0.69) | 0.002 |
| NPTX1 | 59 | 0.89 (0.54, 1.47) | 0.646 |

**Appendix S7. MR results for proteins epidemiologically associated with IPAH**

**Supplementary Table 10-1. Significant MR results for proteins epidemiologically associated with IPAH incidence**

| **Protein** | **Protein source** | **Outcome** | **nsnp** | **OR (95%CI)** | **p-value** | **I^2^** | **p-value (intercept)** |
| --- | --- | --- | --- | --- | --- | --- | --- |
| EDN1 | UK Biobank | GCST007228 | 3 | 1.62 (1.17, 2.25) | 0.004 | < 0.001 | 0.996 |
| ANXA2 | UK Biobank | GCST007228 | 6 | 0.81 (0.71, 0.94) | 0.004 | < 0.001 | 0.448 |
| QSOX1 | UK Biobank | finn-b-I9_HYPTENSPUL | 6 | 2.12 (1.17, 3.86) | 0.014 | < 0.001 | 0.418 |
| QSOX1 | UK Biobank | finn-b-I9_HYPTENSPUL_EXNONE | 6 | 2.10 (1.15, 3.83) | 0.015 | < 0.001 | 0.432 |
| ANXA2 | Fenland study | GCST007228 | 5 | 0.90 (0.83, 0.99) | 0.023 | < 0.001 | 0.653 |
| THBS2 | Fenland study | GCST007228 | 4 | 0.85 (0.74, 0.98) | 0.024 | 0.193 | 0.474 |
| C7 | UK Biobank | finn-b-I9_HYPTENSPUL | 10 | 1.48 (1.05, 2.07) | 0.024 | 0.255 | 0.848 |
| SLAMF7 | UK Biobank | finn-b-I9_HYPTENSPUL_EXNONE | 5 | 0.59 (0.37, 0.94) | 0.025 | < 0.001 | 0.67 |
| SLAMF7 | UK Biobank | finn-b-I9_HYPTENSPUL | 5 | 0.59 (0.37, 0.94) | 0.025 | < 0.001 | 0.675 |
| IGSF3 | UK Biobank | GCST007228 | 3 | 2.21 (1.10, 4.44) | 0.026 | < 0.001 | 0.845 |
| APOC1 | deCODE study | finn-b-I9_HYPTENSPUL_EXNONE | 3 | 10.48 (1.31, 84.02) | 0.027 | < 0.001 | 0.667 |
| APOC1 | deCODE study | finn-b-I9_HYPTENSPUL | 3 | 10.41 (1.29, 84.01) | 0.028 | < 0.001 | 0.687 |
| C7 | UK Biobank | finn-b-I9_HYPTENSPUL | 10 | 1.62 (1.05, 2.49) | 0.028 | < 0.001 | 0.791 |
| C7 | UK Biobank | finn-b-I9_HYPTENSPUL_EXNONE | 10 | 1.61 (1.05, 2.48) | 0.029 | < 0.001 | 0.799 |
| SPINT1 | UK Biobank | GCST007228 | 7 | 1.21 (1.01, 1.45) | 0.035 | < 0.001 | 0.987 |
| LTBP2 | UK Biobank | finn-b-I9_HYPTENSPUL_EXNONE | 5 | 8.95 (1.10, 73.05) | 0.041 | 0.068 | 0.7 |
| LTBP2 | UK Biobank | finn-b-I9_HYPTENSPUL | 5 | 8.34 (1.07, 64.96) | 0.043 | 0.024 | 0.719 |
| NPTX1 | deCODE study | finn-b-I9_HYPTENSPUL | 7 | 2.47 (1.02, 5.98) | 0.045 | < 0.001 | 0.461 |
| ANXA2 | Fenland study | GCST007228 | 5 | 0.90 (0.81, 1.00) | 0.047 | 0.034 | 0.611 |
| NPTX1 | deCODE study | finn-b-I9_HYPTENSPUL_EXNONE | 7 | 2.45 (1.01, 5.92) | 0.048 | < 0.001 | 0.458 |

**Supplementary Table 10-2: All MR results for proteins epidemiologically associated with IPAH incidence (provided in the *Supplementary_Tables*).**

**Supplementary Table 11-1. Significant MR results for proteins epidemiologically associated with mortality**

| **Protein** | **Protein source** | **Outcome** | **nsnp** | **OR (95%CI)** | **p-value** | **I^2^** | **p-value (intercept)** |
| --- | --- | --- | --- | --- | --- | --- | --- |
| NOS1 | UK Biobank | GCST007228 | 4 | 2.19 (1.44, 3.32) | < 0.001 | < 0.001 | 0.267 |
| EDN1 | UK Biobank | GCST007228 | 3 | 1.62 (1.17, 2.25) | 0.004 | < 0.001 | 0.996 |
| SHISA5 | UK Biobank | GCST007228 | 6 | 1.69 (1.18, 2.44) | 0.005 | < 0.001 | 0.942 |
| CCN3 | UK Biobank | GCST007228 | 6 | 1.42 (1.10, 1.83) | 0.008 | 0.08 | 0.453 |
| COL4A1 | UK Biobank | GCST007228 | 3 | 0.64 (0.46, 0.89) | 0.008 | < 0.001 | 0.52 |
| NOS1 | UK Biobank | finn-b-I9_HYPTENSPUL | 4 | 0.13 (0.03, 0.63) | 0.011 | < 0.001 | 0.773 |
| NOS1 | UK Biobank | finn-b-I9_HYPTENSPUL_EXNONE | 4 | 0.13 (0.03, 0.64) | 0.012 | < 0.001 | 0.79 |
| RSPO1 | deCODE study | finn-b-I9_HYPTENSPUL | 4 | 0.15 (0.03, 0.69) | 0.015 | < 0.001 | 0.622 |
| RSPO1 | deCODE study | finn-b-I9_HYPTENSPUL_EXNONE | 4 | 0.15 (0.03, 0.69) | 0.015 | < 0.001 | 0.633 |
| THBS2 | Fenland study | GCST007228 | 4 | 0.85 (0.74, 0.98) | 0.024 | 0.193 | 0.474 |
| RLN2 | UK Biobank | finn-b-I9_HYPTENSPUL_EXNONE | 4 | 6.60 (1.26, 34.48) | 0.025 | < 0.001 | 0.599 |
| RLN2 | UK Biobank | finn-b-I9_HYPTENSPUL | 4 | 6.57 (1.26, 34.26) | 0.026 | < 0.001 | 0.561 |
| LRRN1 | UK Biobank | finn-b-I9_HYPTENSPUL | 6 | 0.18 (0.04, 0.91) | 0.038 | < 0.001 | 0.483 |
| LTBP2 | UK Biobank | finn-b-I9_HYPTENSPUL_EXNONE | 5 | 8.95 (1.10, 73.05) | 0.041 | 0.068 | 0.7 |
| LTBP2 | UK Biobank | finn-b-I9_HYPTENSPUL | 5 | 8.34 (1.07, 64.96) | 0.043 | 0.024 | 0.719 |
| LRRN1 | UK Biobank | finn-b-I9_HYPTENSPUL_EXNONE | 6 | 0.19 (0.04, 0.95) | 0.043 | < 0.001 | 0.48 |

**Supplementary Table 11-2: All MR results for proteins epidemiologically associated with mortality (provided in the *Supplementary_Tables*).**

**Appendix S8. Detailed information of biological risk stratification**

**Supplementary Fig 5.** **Sex-and cluster-stratified proteomic signatures for preclinical patients.**

**Supplementary Fig 6. Sex-and cluster-stratified proteomic signatures for clinical patients.**

| **Supplementary Table 12. Determination of cluster number** | | | | |
| --- | --- | --- | --- | --- |
| **Preclinical patients without IPAH at baseline** | | | | |
|  | k =1 | k = 2^1^ | k = 3 | k = 4 |
| WSS | 8451.04 | 2180.15 | 1508.64 | 1245.74 |
| Silhouette value | / | 0.61 | 0.49 | 0.38 |
| **Clinical patients with IPAH at baseline** | | | | |
|  | k =1 | k = 2^1^ | k = 3 | k = 4 |
| WSS | 3650.71 | 1089.71 | 735.90 | 565.85 |
| Silhouette value | / | 0.62 | 0.48 | 0.42 |

^1^Number of clusters (i.e., k = 2 for clinical patients and k =2 for preclinical patients) were determined with the maximizing silhouette value and elbow method.

Abbreviations: IPAH, idiopathic pulmonary arterial hypertension; WSS, within-cluster sum of squares.

**Appendix S9. Detailed information of prediction models**

**Supplementary Table 13. Performance of prediction models for IPAH incidence and mortality in the overall cohort (both sexes)**

| **Outcomes** | **Models** | **AUC (95%CI)** | **ΔAUC** | **p-value** | **Brier score** | **Calibration intercept** | **Calibration slope** |
| --- | --- | --- | --- | --- | --- | --- | --- |
| **IPAH incidence** | Clinical | 0.753 (0.724, 0.782) | Reference | - | 0.0088 | -1.662 | 0.504 |
|  | Proteomic | 0.689 (0.657, 0.721) | -0.064 | 0.002 | 0.0693 | -4.224 | 0.329 |
|  | Combined | 0.779 (0.750, 0.808) | 0.026 | 0.047 | 0.0086 | -1.585 | 0.528 |
|  | | | | | | | |
| **Mortality** | Clinical | 0.722 (0.672, 0.772) | Reference | - | 0.2214 | 0.055 | 0.579 |
|  | Proteomic | 0.725 (0.675, 0.775) | 0.003 | 0.931 | 0.2176 | 0.044 | 0.564 |
|  | Combined | 0.768 (0.721, 0.814) | 0.045 | 0.031 | 0.2033 | 0.050 | 0.582 |

**Abbreviations:** IPAH, idiopathic pulmonary arterial hypertension; AUC, area under the curve.

**Supplementary Fig 7. Calibration and decision curve analysis of mortality prediction models in the overall cohort**

**Notes: (A)** Calibration plots of the clinical, proteomic, and combined models. Predicted probabilities were grouped into deciles and plotted against the corresponding observed mortality rates. Error bars represent 95% confidence intervals. The grey dashed line indicates perfect agreement between predicted and observed risks. **(B)** Decision curve analysis comparing the net benefit of the three models across a range of threshold probabilities. The grey dashed line represents the strategy of managing all participants, and the black dotted line represents managing none. The combined model included both clinical variables and the protein panel. Model predictions were obtained from the outer-fold out-of-fold predictions of nested cross-validation.

**Supplementary Table 14.** **Incremental predictive value of the protein panel beyond BNP/NT-proBNP for IPAH incidence and mortality in the overall cohort (both sexes)**

| **Outcomes** | **Models** | **AUC (95%CI)** | **ΔAUC** | **p-value** |
| --- | --- | --- | --- | --- |
| **IPAH incidence** | BNP + NT-proBNP | 0.730 (0.694, 0.766) | Reference | - |
|  | Proteomic | 0.689 (0.657, 0.721) | −0.041 | 0.037 |
|  | BNP + NT-proBNP + Proteomic | 0.724 (0.693, 0.755) | −0.006 | 0.733 |
|  |  |  |  |  |
| **Mortality** | BNP + NT-proBNP | 0.583 (0.526, 0.639) | Reference | - |
|  | Proteomic | 0.725 (0.675, 0.775) | 0.142 | < 0.001 |
|  | BNP + NT-proBNP + Proteomic | 0.726 (0.676, 0.776) | 0.143 | < 0.001 |

**Abbreviations:** IPAH, idiopathic pulmonary arterial hypertension; AUC, area under the curve.
